# Influence of lifestyles on physical, psychological, and cognitive co-morbidity among older adults with diabetes in rural area

**DOI:** 10.3389/fpubh.2025.1576697

**Published:** 2025-07-03

**Authors:** Minfu Bai, Yudong Miao, Jingming Wei, Zhanlei Shen, Dongfang Zhu, Jingbao Zhang, Junwen Bai, Ruizhe Ren, Dan Guo, Clifford Silver Tarimo, Jiajia Zhang, Jinxin Cui, Xinran Li, Wenyong Dong, Qiuping Zhao, Mingyue Zhen

**Affiliations:** ^1^Hypertension Department, Fuwai Central China Cardiovascular Hospital, Central China Fuwai Hospital of Zhengzhou University, Zhengzhou, China; ^2^Department of Health Management, College of Public Health, Zhengzhou University, Zhengzhou, China; ^3^Institute of Mental Health, Peking University Sixth Hospital, Beijing, China; ^4^Department of Neurology, Henan Provincial People’s Hospital, People’s Hospital of Zhengzhou University, Zhengzhou, China; ^5^Department of Science and Laboratory Technology, Dar es Salaam Institute of Technology, Dar es Salaam, Tanzania; ^6^Department of Hypertension, Henan Provincial People’s Hospital, People’s Hospital of Zhengzhou University, Zhengzhou, China; ^7^Henan Key Laboratory for Health Management of Chronic Diseases, Central China Fuwai Hospital, Central China Fuwai Hospital of Zhengzhou University, Zhengzhou, China

**Keywords:** lifestyles, physical co-morbidity, psychological co-morbidity, cognitive comorbidity, older adults, diabetes

## Abstract

**Background:**

The association between lifestyles and the co-occurrence of physical, psychological, and cognitive conditions in older adults living with diabetes, especially in rural settings, remains unclear. This study investigated the prevalence of co-morbidity and their association with lifestyle in a rural population of older adults with diabetes.

**Methods:**

From 1st July to 31 August 2023, a cross-sectional study based on the whole cluster sampling method was conducted in Jia County, Henan Province, China. Participants included adults aged ≥65 years. Lifestyle factors assessed included physical activity, diet, smoking, sleep, and social participation. Physical disease was defined as the presence of one or more chronic conditions, while psychological and cognitive disorders were measured using validated scales. Co-morbidity refers to the occurrence of physical, psychological, cognitive diseases. Depending on the number of diseases, we define different comorbidity status. The subgroups of subdivision included eight categories: no co-morbidity, physical co-morbidity, psychological co-morbidity, cognitive co-morbidity, and their four combinations. Logistic regression models were employed to estimate the association between lifestyles and co-morbidity. The net difference in lifestyle between co-morbidity categories was determined using the propensity score matching (PSM).

**Results:**

Among 6057 participants, the overall prevalence of physical, psychological, and cognitive co-morbidity was 86.08%. Regular physical activity, adequate sleep, healthy diet, and active social participation were associated with lower prevalence of co-morbidity. A one-unit increase in the lifestyle score was associated with a 34% (OR: 0.66, 95%CI: 0.61–0.72) reduction in the odds of physical-psychological-cognitive co-morbidity. PSM analyses showed significant differences in lifestyle factors and scores across different co-morbidity status.

**Conclusion:**

Given the higher prevalence of co-morbidity in rural area and the positive association of lifestyle with co-morbidity status, multifactorial lifestyle interventions should be prioritized within diabetic populations to reduce the risk and burden of co-occurring conditions.

## 1 Introduction

The continuous rise in the prevalence of diabetes has become an increasingly serious challenge to contemporary public health. Global systematic review evidence predicts that diabetes cases may rise to 1.3 billion by 2050 (1). Epidemiological surveys show that China has the world’s largest diabetes population, with more than 118 million people living with diabetes, accounting for approximately 22% of all diabetes worldwide (2). The occurrence of diabetes increases the risk of physical impairment (3), disability and death for patients (4, 5). It also adds to the health economic burden (6), raises the risk of Catastrophic household expenditures (7), and poses a serious challenge (8) to traditional single-disease models of public health and health systems. A systematic review from low and middle-income countries showed that the average annual costs (direct and indirect) per person for treating type 2 diabetes ranged from US $29.91 to US $237.38 (9).

Co-morbidity refers to one or more disease states that coexist with or are independent of the primary disease. It is more common for people with diabetes to have two or more conditions, with approximately 80% of diabetic population experiencing co-morbidity (10). This is a significantly higher risk compared to the general population, and the number of diseases tends to increase with age (11, 12). Specifically, the Co-morbidity group was two to three times more likely to suffer from depression compared to general population (13), and had a 1.38% increased relative risk of cognitive disorder than in those with one or no chronic condition (14, 15). The current study shows that the prevalence of diabetes is increasing rapidly (16) and the harm is more serious in rural area than urban area. For example, a comparative study of urban–rural differences showed that rural patients had worse diabetes quality outcomes than urban patients (17). Therefore, it is necessary to explore the prevalence co-morbidity of diabetes in rural area. Many guidelines and studies consistently recommend lifestyle interventions for prevention and diabetes. Extensive research has shown that healthy lifestyles are significantly associated with the prevalence of diabetes co-morbidity. For example, one study showed that low levels of physical activity were associated with a 45% increased risk of diabetes co-morbidity (11) and effectively improve the quality of life of the older adult (18, 19). A longitudinal study shows that physical activity counteracts genetic susceptibility to cognitive function in diabetic populations (20). Healthy lifestyles effectively influence the incidence of diabetes co-morbidity, with positive implications for population health and disease management (21). Although lifestyles and specific patterns of co-morbidity varied between studies (22), these studies did not characterize different combinations of physical, psychological, and cognitive disorders. This limitation is critical because different lifestyles may be associated with specific patterns of co-morbidity prevalence and population.

Given that most countries are experimenting with lifestyle interventions as a new method to prevent and control diabetes and related co-morbidity, it is important to assess lifestyles and integrate them into public health strategies. This study aimed to examine the prevalence and patterns of physical, psychological, cognitive co-morbidity among older adults with diabetes in rural area, and investigate how lifestyle factors differentially influence health outcomes across distinct co-morbidity conditions.

## 2 Methods

### 2.1 Study design and participants

**Figure 1:**
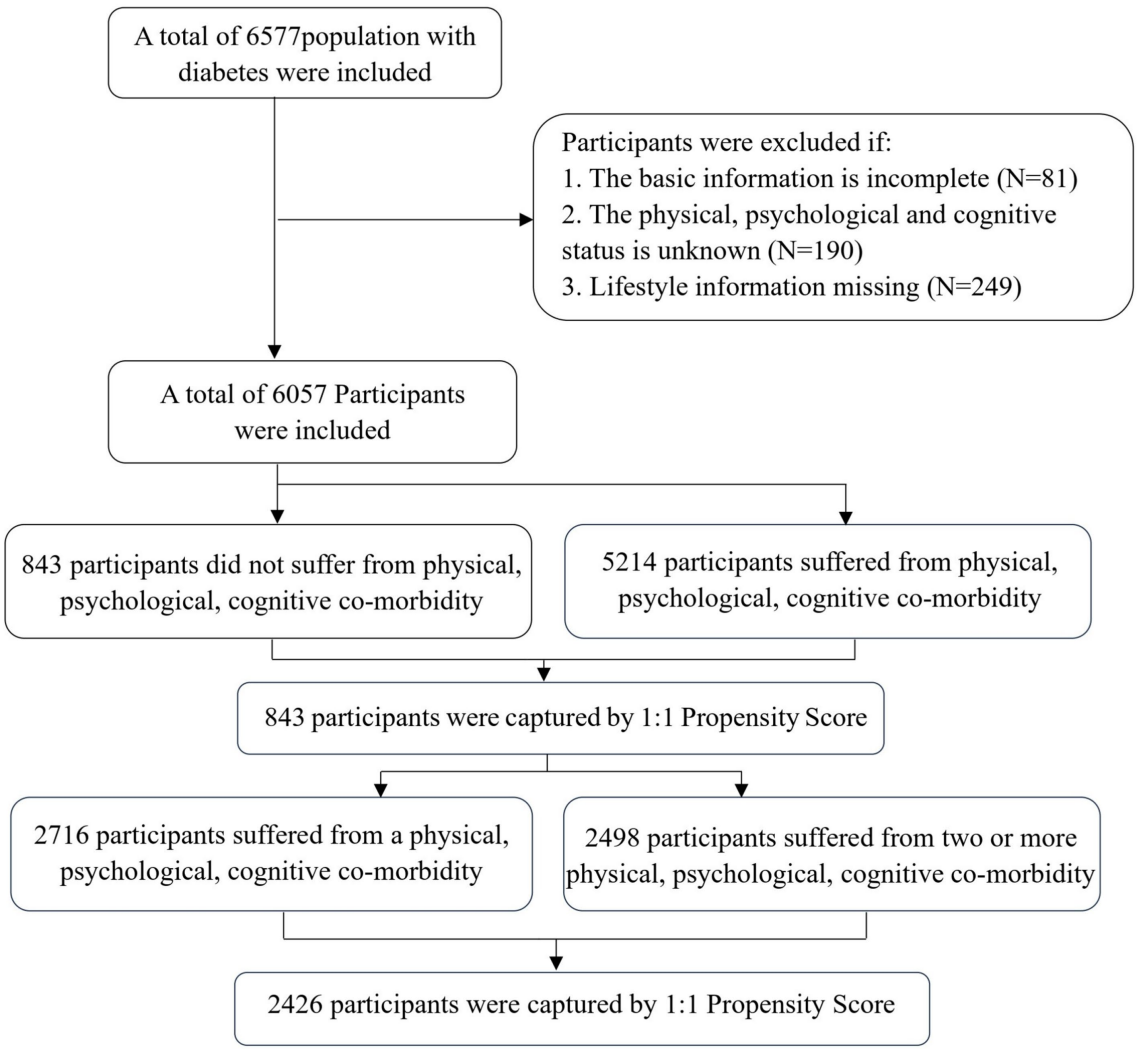
Inclusion and exclusion criteria for study participants.

From 1st July to 31 August 2023, we conducted a cross-sectional survey in Jia County, Henan Province, which is one of the national demonstration area for comprehensive chronic disease prevention and control in China. This study employed a cluster sampling method, with villages as the administrative clustering units, to select adults aged 65 and older with diabetes from the National Basic Public Health Database (NBPHD). The diabetic population was based on blood glucose measurements by primary care providers, and the normal blood glucose value was <7 mmol/L or postprandial blood glucose must be <10 mmol/L. Data collected included basic demographic characteristics, information on healthy lifestyle practices, and physical examination findings. A total of 6,577 questionnaires were collected. Participants were excluded if: (1) The basic information was incomplete; (2) The physical, psychological and cognitive status was unknown; (3) Lifestyle information missing (Figure 1). Each participant signed a written informed consent prior to participating in the study. A total of 6,057 diabetic patients were included, and the effective rate was 92.09%. The study protocol was approved by the Zhengzhou University Medical Ethics Committee (number: 2023-318).

### 2.2 Assessment of lifestyles

The study included five lifestyle-related factors (physical activity, diet, smoking, sleep, and social participant). Physical Activity was assessed using the International Physical Activity Questionnaire (IPAQ). Total physical activity was assessed based on minutes per week of moderate-intensity activity. Regular physical activity was defined as engaging in at least 150 min/week of moderate-intensity activity or 75 min/week of vigorous-intensity activity, or an equivalent combination thereof (23). The food consumption data were collected at baseline based on a food frequency questionnaire including seven major food groups-Vegetables, fruits, (Shell) fish, Processed meats, Unprocessed meats, Whole grains, Refined grains. The responses for each food group were recorded as “every day” or “4–6 days/week” or “1–3 days/week” or “several times/months” or “rarely or never.” Based on the Dietary Priorities for Cardiometabolic Disease, we considered a healthy diet as including at least 4 out of 7 per week, including increased intake of fruits, vegetables, whole grains, and fish, and decreased intake of refined grains and processed or unprocessed meats (24, 25). The smoking status (Non-smoking, former smoking, current smoking) was assessed by self-report. Non-smoking or quitting smoking for ≥30 years was classified as a healthy lifestyle (26). Sleep quality was collected using Pittsburgh sleep quality index scale (PSQI). Sleeping 7–8 h per night was defined as healthy sleep. For social participation, we assessed the frequency of participation in nine different activities, with a frequency score of 2 for “almost every day,” 1 for “occasionally,” and 0 for “rarely or never.” These nine activities were summed and further standardized on a scale of 0 to 1 to produce a total social participation score (27) (Supplementary Table S1).

Regular physical activity, a healthy diet, not smoking or having quit smoking for more than 30 years, adequate sleep, a healthy diet and active social participation were defined as healthy lifestyles. The various lifestyle scores ranged from 0 to 5, with higher scores indicating greater adherence to an overall healthy lifestyle. Because the extreme group with a score of 5 had a smaller number of participants, this group was combined into one category.

### 2.3 Assessment of co-morbidity

Participants were identified as having a physical disease if they self-reported having one of the following four major categories of chronic disease: cardiovascular disease (coronary heart disease, stroke, and hypertension), respiratory disease (tuberculosis, asthma, and chronic obstructive pulmonary disease), chronic kidney disease, and arthritis. Psychological disease was measured using anxiety and depression scales (General Anxiety Disorder-7 and Patient Health Queationnaire-9) (28). The total score was the sum of the individual item scores, with a score of 5 or less indicating no depression or anxiety. The Chinese versions of GAD-7 and PHQ-9 have been widely used and well validated in several studies, and showed good internal consistency in the current study. Those who suffer from anxiety or depression were recognized as having a psychological disease. Cognitive disorder was scored using the Mini-Mental State Examination (MMSE) scale, with each item scoring 1 point for correct response and 0 points for incorrect or no idea. The classification of cognitive disorder was linked to the level of education, with a score below 17 for the illiterate population, below 20 for those with the primary school education, and below 24 for those with junior high school education or above, were considered indicative of a cognitive disorder.

Co-morbidity refers to the occurrence of physical, psychological, cognitive diseases. Based on the number of diseases suffered, we have defined different comorbidity status, which can be divided into no co-morbidity, with one co-morbidity condition, and with more than two co-morbidity conditions. Based on the different combinations of the physical, psychological, cognitive, we defined the outcome variables as eight subgroups. The eight subgroups include physical co-morbidity, psychological co-morbidity, cognitive co-morbidity, physical-psychological (P-Ps) co-morbidity, physical-cognitive (P-C) co-morbidity, psychological-cognitive (Ps-C) co-morbidity, physical-psychological-cognitive (P-Ps-C) co-morbidity, and No condition (not suffering from any of these).

### 2.4 Assessment of covariates

We obtain basic information through face-to-face interviews and Public Health databases conducted by trained professionals. Basic information mainly included: (1) demographic characteristics: sex, age, education level, marital status, and occupation; (2) disease status: duration of disease, complication, make medication; (3) physical examination indicators: height and weight. Height and weight were measured using specialized tools, with the average of three measurements taken to calculate the body mass index (BMI).

### 2.5 Statistical analysis

Categorical variables were expressed as frequencies and percentages. Chi-squared tests (categorical variables) and Wilcoxon rank-sum tests (continuous variables) were used to assess differences in the distribution of baseline information, lifestyle factors, and other covariates between groups. Using Venn diagram describes the participants proportion distribution of physical, psychological, cognitive co-morbidity. Multivariable-adjusted logistic regression models were employed to estimate the association between different lifestyles and the defined outcomes. Adjusted regressions included sex, age, education, marital status, occupation, disease duration, complication, medication use, and BMI. Propensity score matching (PSM) was used to balance the distribution of covariates between participants with and without co-morbidity. The nearest neighbor 1:1 matching method (caliper value = 0.05) was used to ensure that the propensity score difference between matching pairs was <0.05 log standard deviation. Following 1:1 matching based on propensity scores, 843 participants were included in both the no-co-morbidity group and the combined co-morbidity group, and 2,426 participants were included in the analysis of different co-morbidity statuses. All analyses were performed using R 4.3.2 and Stata 17.0. Tests were two-tailed and *p* < 0.05 was considered statistically significant.

## 3 Results

### 3.1 Prevalence of co-morbidity and basic characteristics of the participants

A total of 6,057 older adults with diabetes completed the survey and were included for further analysis, 63.89% of whom were women. Table 1 shows the prevalence of co-morbidity among the participants. The overall prevalence of physical, psychological, and cognitive co-morbidity was 86.08% (Table 1). In brief, sex, age, marital status, duration of disease, complication, take medicine were significantly different between groups. There are clear differences between different lifestyles, except for diet. The study found that the prevalence of one co-morbidity was 44.84%, and having more than two diseases was 41.24%. There were 9.81% of diabetic patients with physical, psychological, cognitive co-morbidity. In addition to marital status, we found significant differences in co-morbidity among participants. There are significant differences among different lifestyles (Supplementary Table S2).

**Table 1:** The prevalence of co-morbidity and basic characteristics of the participants.

Variables	Total (%)	Co-morbidity (%)	No co-morbidity (%)	*p*
Population	6,057 (100.00)	5,214(86.08)	843 (13.92)	
Sex				*<0.001*
Male	2,187 (36.11)	1806 (34.64)	381 (45.20)	
Female	3,870 (63.89)	3,408 (65.36)	462 (54.80)	
Age				*0.005*
65–70	2,544 (42.00)	2,143 (41.10)	401 (47.57)	
71–80	1927 (31.81)	1,678 (32.18)	249 (29.54)	
81–90	1,080 (17.83)	947 (18.16)	133 (15.78)	
≥ 91	506 (8.35)	446 (8.55)	60 (7.12)	
Marital status				*0.011*
Unmarried	160 (2.64)	135 (2.59)	25 (2.97)	
Married	4,521 (74.64)	3,861 (74.05)	660 (78.29)	
Widowed	1,376 (22.72)	1,218 (23.36)	158 (18.74)	
Education				*0.102*
Illiteracy	2,696 (44.51)	2,334 (44.76)	362 (42.94)	
Primary school	1927 (31.81)	1,659 (31.82)	268 (31.79)	
Junior high school	1,028 (16.97)	888 (17.03)	140 (16.61)	
Senior high school and above	406 (6.70)	333 (6.39)	73 (8.66)	
Occupation				*0.166*
Agriculture	3,775 (62.32)	3,238 (62.10)	537 (63.70)	
Non-agriculture	1890 (31.20)	1,647 (31.59)	243 (28.83)	
Retirement	392 (6.47)	329 (6.31)	63 (7.47)	
Duration of disease				*0.001*
≤5 years	2092 (34.54)	1752 (33.60)	340 (40.33)	
6–10 years	1,452 (23.97)	1,269 (24.34)	183 (21.71)	
11–15 years	1,347 (22.24)	1,173 (22.50)	174 (20.64)	
16–20 years	681 (11.24)	585 (11.22)	96 (11.39)	
≥21 years	485 (8.01)	435 (8.34)	50 (5.93)	
Complication				*<0.001*
Yes	3,250 (53.66)	2,898 (55.58)	352 (41.76)	
No	2,807 (46.34)	2,316 (44.42)	491 (58.24)	
BMI				*<0.001*
18.5–23.9	2,190 (36.16)	1830 (35.10)	360 (42.70)	
>23.9	3,664 (60.49)	3,219 (61.74)	445 (52.79)	
<18.5	203 (3.35)	165 (3.16)	38 (4.51)	
Take medicine				*0.015*
No	477 (7.88)	393 (7.54)	84 (9.96)	
Yes	5,580 (92.12)	4,821 (92.46)	759 (90.04)	
Physical activity				*<0.001*
No exercise	1731 (28.58)	1,560 (29.92)	171 (20.28)	
Exercise	4,326 (71.42)	3,654 (70.08)	672 (79.72)	
Diet				*0.197*
Unhealthy	4,856 (80.17)	4,194 (80.44)	662 (78.53)	
Healthy	1,201 (19.83)	1,020 (19.56)	181 (21.47)	
Smoking				*<0.001*
Current smoking	1,255 (20.72)	1,038 (19.91)	217 (25.74)	
No-Smoking	4,802 (79.28)	4,176 (80.09)	626 (74.26)	
Sleep				*0.001*
<7 h/>8 h	2,333 (38.52)	2050 (39.32)	283 (33.57)	
7–8 h	3,724 (61.48)	3,164 (60.68)	560 (66.43)	
Social participation				*<0.001*
No	2,880 (47.55)	2,530 (48.52)	350 (41.52)	
Yes	3,177 (52.45)	2,684 (51.48)	493 (58.48)	

Co-morbidity refers to the occurrence of physical, psychological, cognitive diseases; P for Chi-square test.

**Figure 2:**
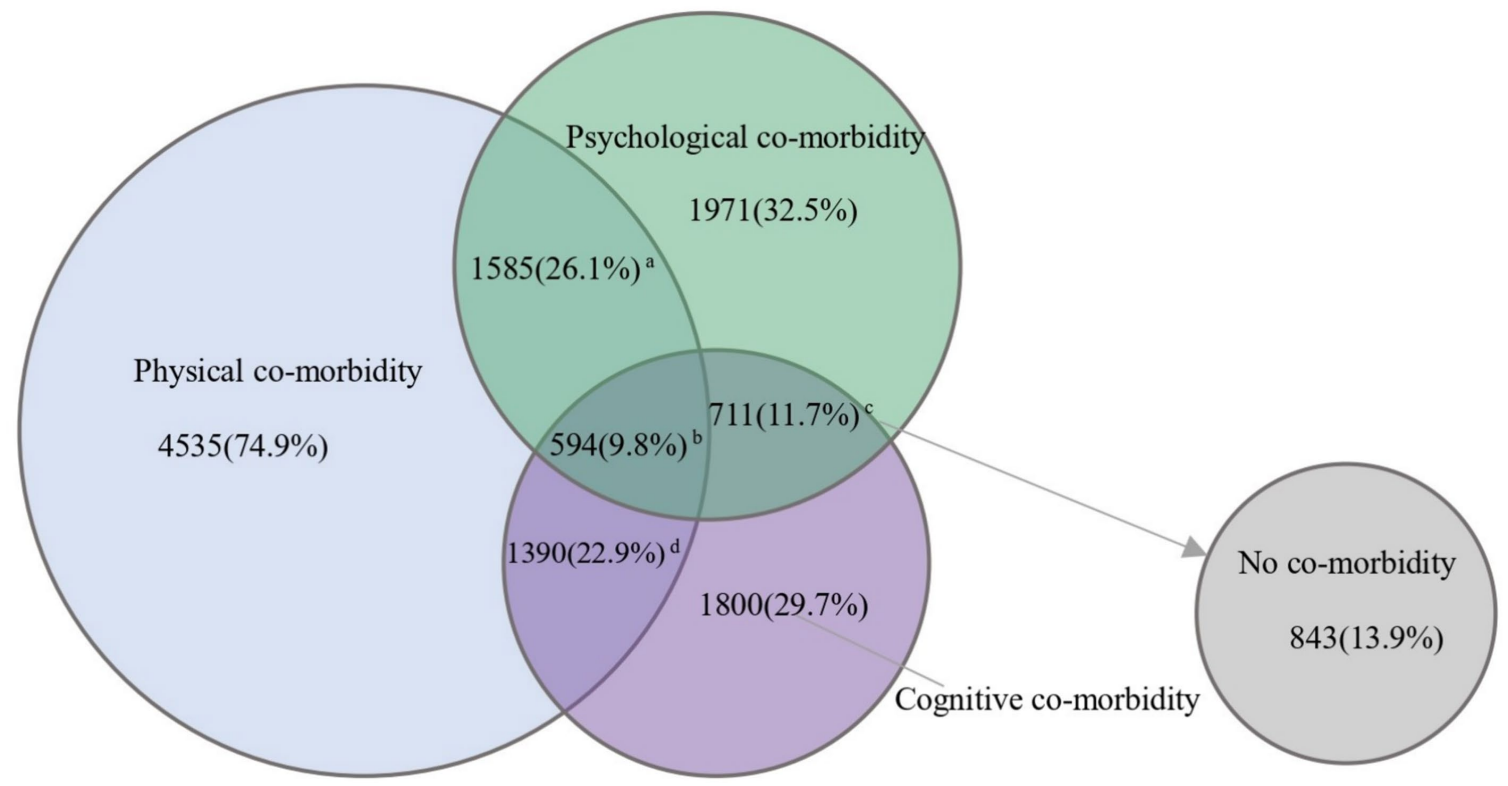
Venn diagram of the prevalence of different co-morbidity patterns. “a” indicates physical-psychological co-morbidity; “b” indicates physical-psychological-cognitive co-morbidity; “c” indicates psychological-cognitive co-morbidity; “d” for physical-cognitive co-morbidity.

Among the different subgroups, the prevalence of physical co-morbidity, psychological co-morbidity, and cognitive co-morbidity among participants was 74.9%, 32.5%, and 29.7%, respectively; the prevalence of P-Ps co-morbidity was 26.1%, P-C co-morbidity was 22.9%, Ps-C co-morbidity was 11.7%, and P-Ps-C co-morbidity was 9.8% (Figure 2). There were significant differences in lifestyle among different subgroups. Sex differences were found in most subgroups, but not in P-C co-morbidity, as was the case with lifestyle diets. Overall, there are significant differences in lifestyles among the subgroups. For example, the prevalence of P-Ps-C co-morbidity is lower in people who are moderately physically active (8.25%, 95%CI:7.47–9.11; 13.69%, 95%CI:12.15–15.39) (All *p* < 0.05). Full prevalence results can be found in the appendix (Supplementary Tables S3–S5).

### 3.2 Association between different lifestyles and co-morbidity

**Figure 3:**
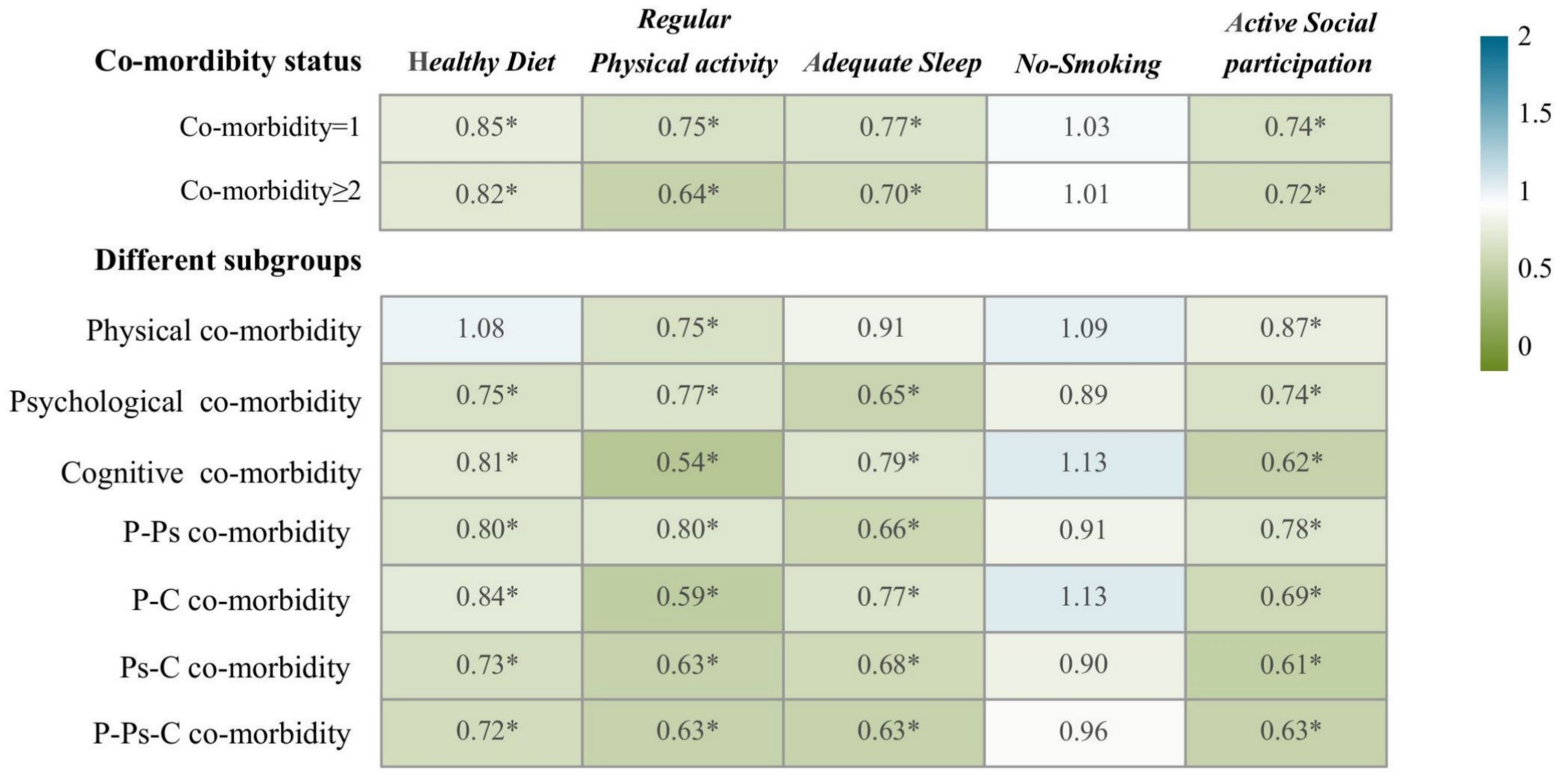
The different lifestyles in relation to co-morbidity status and patterns. *A meaningful value for the regression result. Comorbidity status refers to the number of physical, psychological, cognitive diseases suffered. P-Ps co-morbidity for physical-psychological co-morbidity; P-C co-morbidity for physical-cognitive co-morbidity; Ps-C co-morbidity for psychological-cognitive co-morbidity; P-Ps-C co-morbidity for physical-psychological-cognitive co-morbidity. Adjusted OR incorporated sex, age, education, marital status, occupation, disease duration, complication, medication use, and BMI.

Figure 3 shows the effect of different lifestyles on the prevalence of co-morbidity and different subgroups in the participants. Regular physical activity, adequate sleep, and active social participation were associated with lower prevalence of co-morbidity in adjusted OR. Among participants with more than two co-morbidity conditions, different lifestyles had a positive protective effect on co-morbidity (OR: 0.82, 95%CI:0.71–0.94; OR:0. 0.64, 95%CI:0.57–0.72; OR:0.70, 95%CI:0.63–0.78; OR:0.72, 95%CI:0.64–0.80). Across the different subgroups, we found that physical activity played a notable protective role, and that diet, sleep, and social participation also had a major impact on the outcomes. Significant effects of physical activity and regular physical activity on physical disease after adjusted OR (OR:0.75, 95%CI:0.65–0.861; OR:0.87, 95%CI:0.77–0.98). However, after adjusting for confounding factors, smoking status did not seem to affect the outcome variables. In the study, we also found that diet had no effect on people without co-morbidity and people with physical diseases.

### 3.3 Association between lifestyle scores and co-morbidity

The distribution of lifestyle scores was concentrated at two and three points, with a mean score of 3. Participants in the Ps-C co-morbidity group exhibited the lowest percentage of all participants, achieving the highest possible score of 5. In addition, there was a clear downward trend in lifestyle scores as the number of diseases increased (Supplementary Figure S1). We also examined the association between different lifestyle scores and basic population characteristics, finding significant differences in sex, age, marital status, education, and occupation (*p* < 0.05) (Supplementary Table S6).

**Figure 4:**
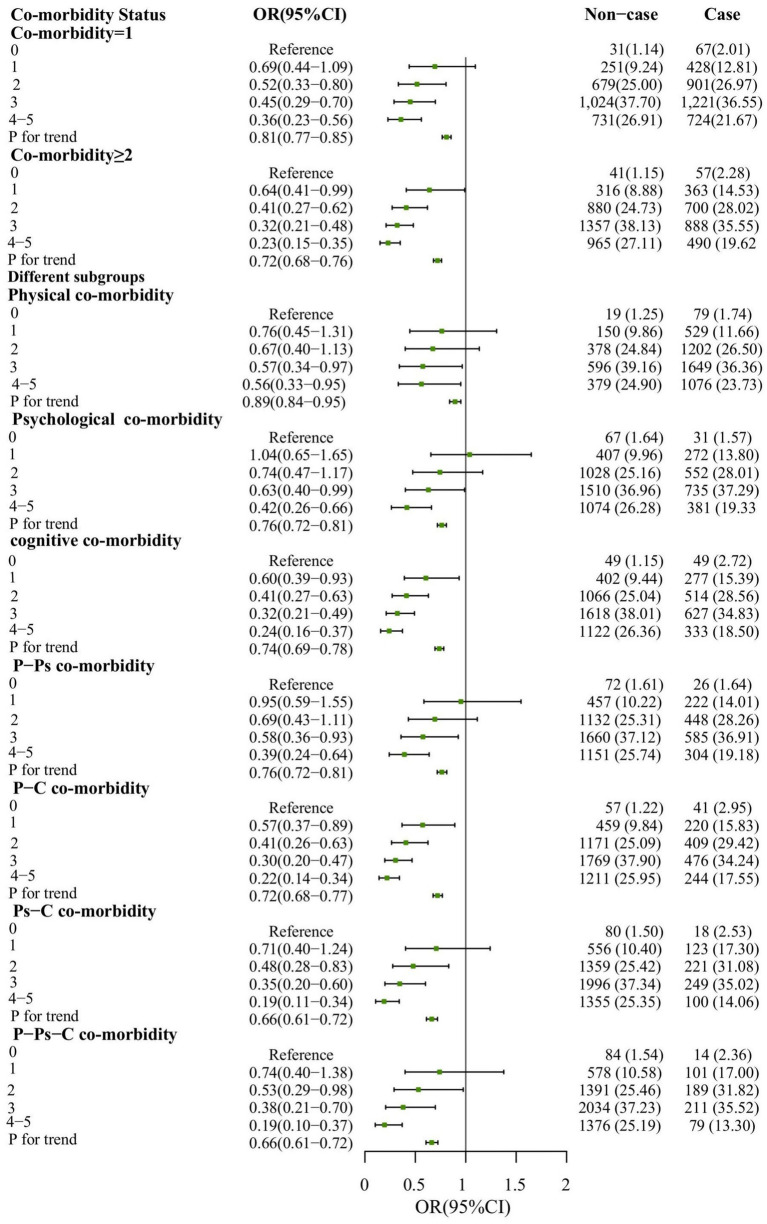
The regression results for lifestyle scores and co-morbidity. Comorbidity status refers to the number of physical, psychological, cognitive diseases suffered. P-Ps co-morbidity for physical-psychological co-morbidity; P-C co-morbidity for physical-cognitive co-morbidity; Ps-C co-morbidity for psychological-cognitive co-morbidity; P-Ps-C co-morbidity for physical-psychological-cognitive co-morbidity. Adjusted OR incorporated sex, age, education, marital status, occupation, disease duration, complication, medication use, and BMI.

Adjusted regression indicated that an increase in lifestyle score was effective in reducing the risk of co-morbidity prevalence (OR: 0.89, 95%CI:0.44–1.81; OR: 0.62, 95%CI:0.31–1.22; OR: 0.50, 95%CI:0.25–0.98; OR: 0.42, 95%CI:0.21–0.83). The risk of co-morbidity is reduced by 20% for every unit increase in lifestyle score (OR:0.80, 95%CI:0.74–0.86). After adjusting for confounders, an increase in lifestyle score was significantly associated with a reduced risk of different subgroups (*P*-trend <0.001). For a unit increase in lifestyle score, the risk of prevalence of P-Ps co-morbidity was reduced by 24% (OR: 0.76, 95% CI: 0.72–0.81), the risk of prevalence of P-C co-morbidity was decreased by 28% (OR: 0.72, 95% CI: 0.68–0.77), and the risk of prevalence of Ps-C diseases was reduced by 34% (OR: 0.66, 95% CI: 0.61–0.72). 34% reduction in risk of prevalence of P-Ps-C co-morbidity was also observed (OR: 0.66, 95% CI: 0.61–0.72) (Figure 4). And, based on the lifestyle means, we found that there is still a strong relationship between lifestyle category and co-morbidity (Supplementary Table S7).

### 3.4 The PSM results of lifestyle differences in co-morbidity

**Figure 5:**
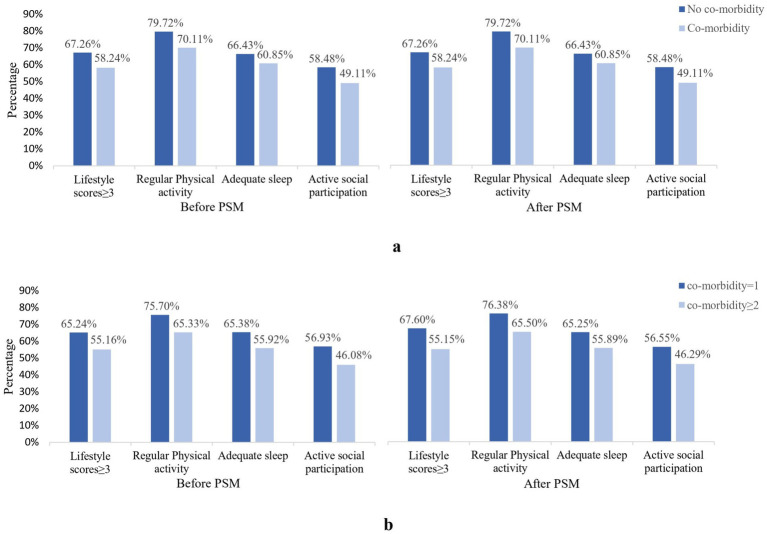
Percentage of healthy lifestyle between different co-morbidity status before and after PSM. **(a)** Refers to lifestyle differences between no co-morbidity and co-morbidity, matched sex, age, marital status, duration of disease, complication, take medicine, BMI. **(b)** Refers to lifestyle differences between co-morbidity status, matched sex, age, education, duration of disease, complication, take medicine.

PSM was used for homogenization to observe the lifestyle of participants with different co-morbidity status. 843 participants were included in each group (no co-morbidity and any co-morbidity), selected from a total of 6,057 participants. After PSM for sex, age, marital status, duration of illness, complications, medication, and BMI, we found no statistically significant differences between no condition and co-morbidity participants in all covariates (all *p* > 0.05) (Supplementary Tables S8, S9 and Supplementary Figure S2) Based on PSM results, we find the proportion of the mean lifestyle score in the no condition participants was still 9.02% higher than that in the co-morbidity group. The proportion of different lifestyle factors was significantly different (Figure 5a). Similarly, a total of 2,426 samples from 6,057 participants were matched using PSM across the different co-morbidity groups (Supplementary Tables S10, S11 and Supplementary Figure S3). We find the proportion of lifestyle score in participants with fewer co-morbidity was still 11.08% higher than that in participants with more co-morbidity. The proportion of different lifestyle factors was significantly different (Figure 5b).

Within the subgroups by sex, the associations between lifestyle and most outcomes persisted. Lifestyle continues to have a positive protective effect against co-morbidity. However, we also found that the protective effect of lifestyle was more pronounced in male (Supplementary Table S12). In addition, we excluded the group over 80 years old to verify the robustness and performed sensitivity analysis, which found that the lifestyle score remained a positive protective effect on co-morbidity (Supplementary Table S13).

## 4 Discussion

The current study involved older adults with diabetes in rural area to assess the prevalence of physical, psychological, cognitive co-morbidity and the impact of lifestyle factors. Among older adults with diabetes, the overall prevalence of co-morbidity was 86.08%. Within this group, 9.8% experienced all three types of co-morbidity. Only 13.9% of the diabetic population had no reported co-morbidity. These prevalence rates are much higher than those observed in the general population (29). This may be due to the fact that the population we surveyed was predominantly older adult from rural area and all were diabetic population (30). In addition, the prevalence of co-morbidity was observed to be much higher in women than in men (31). The reasons for the development of each disease and the damage it causes are not the same (32), which means that prevention and treatment must be more targeted and tailored to the needs of the specific population.

Strong correlations were found between co-morbidity and key lifestyle factors. Our study reveals that regular physical activity is a crucial protective factor against the development of physical, psychological and cognitive co-morbidity among older adults with diabetes. A systematic review of 128,119 subjects showed that physical activity was highly beneficial in improving depression and anxiety, both in the general population and in patients with chronic diseases (33). Although rural settings may provide certain advantages for engaging in physical activity, our study highlights the substantial positive impact of physical activity on reducing co-morbidity prevalence among older adults with diabetes. In this regard, we advocate for the prioritization of physical activity as a fundamental element of lifestyle guidance, rather than simply a supportive element. In addition to traditional healthy lifestyles, emerging lifestyles are gaining attention (34, 35). In a review of co-morbidity, emerging healthy lifestyles, such as sleep and active social participation, were found to play an important role in chronic disease co-morbidity (36). Therefore, we can focus on adopting different lifestyle guidelines for different regions and populations in order to better reduce incidences of co-morbidity. Smoking and diet were not associated with co-morbidity. One possibility is that most of the surveyed population did not smoke or had quit smoking for many years, and there was a survivor effect. Diet may be attributed to the fact that the diet structure of the older adult population in rural areas is relatively simple, and there is no significant difference. In addition, we found that there was indeed a net difference in the lifestyle under different co-morbidity status, suggesting that lifestyle can be an important component of primary prevention in rural area.

Although a single lifestyle also had a positive effect on co-morbidity, the protective effect against co-morbidity was substantial with increasing lifestyle. This may be because the beneficial effects of lifestyle are synergistic and cumulative, and the synergistic associations of these factors are greater than the individual effects (37). Our findings emphasize the importance of the combined health effects of different lifestyles in preventing physical, psychological and cognitive co-morbidity among older adults with diabetes, rather than a single lifestyle choice. Therefore, we need to place more emphasis on the combined effects of multiple lifestyles in our efforts to improve disease prevention and expand disease control. There are significant differences in lifestyle among patients with different co-morbidity status, and lifestyle has an obvious protective effect, which suggests that as a special population of diabetes, it is necessary to strengthen the primary prevention of lifestyle and increase health awareness.

This study has the following advantages: Based on county level, cluster sampling will provide evidence and replicable experience for chronic disease co-morbidity management in county level. Previous studies have focused on the general population or a single disease. We are the first to examine the relationship between physical, psychological, cognitive co-morbidity and lifestyle factors by excluding confounding variables through PSM.

There are also some limitations to this study. First, the cross-sectional nature of this study prevented us from assessing longitudinal effects of lifestyle. Although PSM was used in this study to avoid the effects of some confounding variables, it is still not comprehensive enough. Second, psychological, and cognitive co-morbidity were self-reported by respondents, which may introduce recall bias and omit undiagnosed illnesses. In addition, the classification of physical diseases is still not comprehensive. However, it is worth noting that previous studies have shown a high correlation between self-reported histories of physical and cognitive disorders and electronic health records. Third, the lifestyle scores in this study were derived from the sum of scores for lifestyle-related factors. We hypothesized that these lifestyle factors would have the same effect on physical status; however, this may mask a true interaction between lifestyle factors. Fourth, there may be potential confounders that could confound our results, such as other socioeconomic status and access to health care. These confounding factors can also affect the lifestyle of people with diabetes, which in turn affects co-morbidity.

## 5 Conclusion

In conclusion, this study found a relatively high prevalence of physical, psychological, and cognitive co-morbidity among diabetic older adults in rural area, despite moderate levels of overall healthy lifestyle practices. Given the observed association between higher lifestyle scores and reduced co-morbidity risk, comprehensive lifestyle interventions, rather than focusing on individual behaviors, should be prioritized for the prevention of diabetes-related co-morbidity. Developing tailored interventions based on prevalent co-morbidity patterns and their association with healthy lifestyles is crucial for enhancing intervention precision and adherence in rural populations.

## Data Availability

The raw data supporting the conclusions of this article will be made available by the authors, without undue reservation.
