# Correction: Influence of lifestyles on physical, psychological, and cognitive co-morbidity among older adults with diabetes in rural area

**DOI:** 10.3389/fpubh.2025.1662805

**Published:** 2025-07-17

**Authors:** Frontiers Production Office

**Affiliations:** Frontiers Media SA, Lausanne, Switzerland

**Keywords:** lifestyles, physical co-morbidity, psychological co-morbidity, cognitive comorbidity, older adults, diabetes

Author Minfu Bai was erroneously assigned to the affiliation “Department of Health Management, College of Public Health, Zhengzhou University, Zhengzhou, China”. This affiliation has now been removed for the author. The affiliation “Hypertension Department, Fuwai Central China Cardiovascular Hospital, Central China Fuwai Hospital of Zhengzhou University, Zhengzhou, China” was omitted for author Minfu Bai. This affiliation has now been added for the author.

The original version of this article has been updated.

