# Supplementary material for: Influence of lifestyles on physical, psychological, and cognitive co-morbidity among older adults with diabetes in rural area
Source: Front Public Health. 2025 Jul 3;13:1576697. doi: 10.3389/fpubh.2025.1576697 (PMC12309496; doi:10.3389/fpubh.2025.1576697)
Supplement: Supplementary file 1 [file Table_1.docx]

**Supplementary Material**

**Table.S1.** Description and scoring criteria of lifestyle factors

**Table.S2**. The prevalence and characteristics of physical, psychological, cognitive co-morbidity status

**Table.S3.** Population distribution in physical, psychological and cognitive co-morbidity among participants

**Table.S4.** Prevalence rate of individual physical co-morbidity, psychological co-morbidity and cognitive co-morbidity of the participants

**Table.S5.** Prevalence rate of four combinations among participants

**Fig.S1.** Stacking plot of lifestyle scores in physical, psychological and cognitive co-morbidity

**Table.S6.** Basic characteristics of lifestyle scores among older adults with diabetes

**Table.S7.** The association of lifestyle category with co-morbidity

**Table.S8.** Balance test of PSM for no co-morbidity and co-morbidity samples

**Fig.S2.** Common support domain of PSM for no co-morbidity and co-morbidity groups

**Table.S9.** Differences in lifestyle among no co-morbidity and co-morbidity after PSM

**Table.S10.** Balance test of PSM for one co-morbidity and two or more co-morbidity samples

**Fig.S3**. Common support domain of PSM for one co-morbidity and two or more co-morbidity groups

**Table.S11.** Differences in lifestyle among co-morbidity status after PSM

**Table.S12.** Subgroup analysis by sex (fully adjusted model)

**Table.S13.** Sensitivity analysis by excluding over 80 years old

| **Table.S1.** Description and scoring criteria of lifestyle factors | | |
| --- | --- | --- |
| **Lifestyle factors** | **Category** | **Score** |
| **Smoking status** | Current non-smoking (never smoking/quit smoking for 30 years) | 1 |
|  | smoking | 0 |
| **Regular Physical activity** | At least 150 minutes/week of moderate-intensity activity, 75 minutes/week of vigorous activity or both equivalent combination | 1 |
| **Adequate Sleep** | To keep 7-8 hours of sleep every day | 1 |
| **Healthy Diet** |  |  |
| Vegetables | Daily, 4-6 days/week, 1-3 days/week | 1 |
| fruits | Daily, 4-6 days/week, 1-3 days/week | 1 |
| (Shell) fish | Daily, 4-6 days/week | 1 |
| Processed meats | 1-3 days/week, several times/month, No/very little | 1 |
| Unprocessed meats | several times/month, No/very little | 1 |
| Whole grains | Daily, 4-6 days/week | 1 |
| Refined grains | 1-3 days/week, several times/month, No/very little | 1 |
| **Social participation** |  |  |
| Housework tasks | Almost everyday/Not every day, but at least once a week | 2 |
|  | Not every week, but at least once a month | 1 |
|  | Not every month, but sometimes/Never | 0 |
| Personal outdoor activities | Almost everyday/Not every day, but at least once a week | 2 |
|  | Not every week, but at least once a month | 1 |
|  | Not every month, but sometimes/Never | 0 |
| Gardening | Almost everyday/Not every day, but at least once a week | 2 |
|  | Not every week, but at least once a month | 1 |
|  | Not every month, but sometimes/Never | 0 |
| Reading | Almost everyday/Not every day, but at least once a week | 2 |
|  | Not every week, but at least once a month | 1 |
| Rearing domestic animals/pets | Almost everyday/Not every day, but at least once a week | 2 |
|  | Not every week, but at least once a month |  |
|  | Not every month, but sometimes/Never |  |
| Playing cards/mahjong | Almost everyday/Not every day, but at least once a week | 2 |
|  | Not every week, but at least once a month | 1 |
|  | Not every month, but sometimes/Never | 0 |
| Watching TV/ listening to the radio | Almost everyday/Not every day, but at least once a week | 2 |
|  | Not every week, but at least once a month | 1 |
|  | Not every month, but sometimes/Never | 0 |
| Attending social activities | Almost everyday/Not every day, but at least once a week | 2 |
|  | Not every week, but at least once a month | 1 |
|  | Not every month, but sometimes/Never | 0 |
| Taking a tour | Almost everyday /Not every day, but at least once a week | 2 |
|  | Not every week, but at least once a month | 1 |
|  | Not every month, but sometimes/Never | 0 |
| Diet: If it contains four types, it is considered healthy. | | |
| Social participation score ranged from 0 to 18, and subsequently converted to a scale from 0 to 1by dividing the maximum possible score. | | |

**Table.S2**. The prevalence and characteristics of physical, psychological, cognitive co-morbidity status

| **Variables** | **Total (%)** | ***Co-morbidity status*** | | | ***P*** |
| --- | --- | --- | --- | --- | --- |
|  |  | Co-morbidity=1 | Co-morbidity=2 | Co-morbidity=3 |  |
| **Population** | 6057 (100.00） | 2716 (44.84) | 1904(31.43) | 594(9.81) |  |
| **Sex** |  |  |  |  | *<0.001* |
| Male | 2187 (36.11) | 1046 (38.51) | 600 (31.51) | 160 (26.94) |  |
| Female | 3870 (63.89) | 1670 (61.49) | 1304 (68.49) | 434 (73.06) |  |
| **Age** |  |  |  |  | *0.003* |
| 65-70 | 2544 (42.00) | 1161 (42.75) | 762 (40.02) | 220 (37.04) |  |
| 71-80 | 1927 (31.81) | 868 (31.96) | 609 (31.99) | 201 (33.84) |  |
| 81-90 | 1080 (17.83) | 469 (17.27) | 369 (19.38) | 109 (18.35) |  |
| ≥ 91 | 506 (8.35) | 218 (8.03) | 164 (8.61) | 64 (10.77) |  |
| **Marital status** |  |  |  |  | *0.060* |
| Unmarried | 160 (2.64) | 72 (2.65) | 52 (2.73) | 11 (1.85) |  |
| Married | 4521 (74.64) | 2009 (73.97) | 1397 (73.37) | 455 (76.60) |  |
| Widowed | 1376 (22.72) | 635 (23.38) | 455 (23.90) | 128 (21.55) |  |
| **Education** |  |  |  |  | *<0.001* |
| Illiteracy | 2696 (44.51) | 1215 (44.73) | 887 (46.59) | 232 (39.06) |  |
| Primary school | 1927 (31.81) | 864 (31.81) | 583 (30.62) | 212 (35.69) |  |
| Junior high school | 1028 (16.97) | 433 (15.94) | 328 (17.23) | 127 (21.38) |  |
| Senior high school and above | 406 (6.70) | 204 (7.51) | 106 (5.57) | 23 (3.87) |  |
| **Occupation** |  |  |  |  | *0.033* |
| Agriculture | 3775 (62.32) | 1684 (62.00) | 1165 (61.19) | 389 (65.49) |  |
| Non-agriculture | 1890 (31.20) | 841 (30.96) | 636 (33.40) | 170 (28.62) |  |
| Retirement | 392 (6.47) | 191 (7.03) | 103 (5.41) | 35 (5.89) |  |
| **Duration of disease** |  |  |  |  | *<0.001* |
| ≤5 years | 2092 (34.54) | 974 (35.86) | 619 (32.51) | 159 (26.77) |  |
| 6-10 years | 1452 (23.97) | 657 (24.19) | 477 (25.05) | 135 (22.73) |  |
| 11-15 years | 1347 (22.24) | 578 (21.28) | 443 (23.27) | 152 (25.59) |  |
| 16-20 years | 681 (11.24) | 300 (11.05) | 207 (10.87) | 78 (13.13) |  |
| ≥21 years | 485 (8.01) | 207 (7.62) | 158 (8.30) | 70 (11.78) |  |
| **Complication** |  |  |  |  | *<0.001* |
| Yes | 3250 (53.66) | 1380 (50.81) | 1149 (60.35) | 369 (62.12) |  |
| No | 2807 (46.34) | 1336 (49.19) | 755 (39.65) | 225 (37.88) |  |
| **BMI** |  |  |  |  | *<0.001* |
| 18.5-23.9 | 2190 (36.16) | 934 (34.39) | 667 (35.03) | 229 (38.55) |  |
| >23.9 | 3664 (60.49) | 1696 (62.44) | 1175 (61.71) | 348 (58.59) |  |
| <18.5 | 203 (3.35) | 86 (3.17) | 62 (3.26) | 17 (2.86) |  |
| **Take medicine** |  |  |  |  |  |
| No | 477 (7.88) | 226 (8.32) | 132 (6.93) | 35 (5.89) |  |
| Yes | 5580 (92.12) | 2490 (91.68) | 1772 (93.07) | 559 (94.11) |  |
| **Physical activity** |  |  |  |  | *<0.001* |
| No exercise | 1731 (28.58) | 694 (25.55) | 629 (33.04) | 237 (39.90) |  |
| Exercise | 4326 (71.42) | 2022 (74.45) | 1275 (66.96) | 357 (60.10) |  |
| **Diet** |  |  |  |  | *0.002* |
| Unhealthy | 4856 (80.17) | 2137 (78.68) | 1555 (81.67) | 502 (84.51) |  |
| Healthy | 1201 (19.83) | 579 (21.32) | 349 (18.33) | 92 (15.49) |  |
| **Smoking** |  |  |  |  | *<0.001* |
| Current smoking | 1248 (20.60) | 599 (22.05) | 340 (17.86) | 92 (15.49) |  |
| No-Smoking | 4809 (79.40) | 2117 (77.95) | 1564 (82.14) | 502 (84.51) |  |
| **Sleep** |  |  |  |  | *<0.001* |
| Others | 2333 (38.52) | 949 (34.94) | 806 (42.33) | 295 (49.66) |  |
| 7-8 hours | 3724 (61.48) | 1767 (65.06) | 1098 (57.67) | 299 (50.34) |  |
| **Social participation** |  |  |  |  | *<0.001* |
| No | 2880 (47.55) | 1183 (43.56) | 988 (51.89) | 359 (60.44) |  |
| Yes | 3177 (52.45) | 1533 (56.44) | 916 (48.11) | 235 (39.56) |  |
| P for Chi-square test |  |  |  |  |  |

| **Table.S3.** Population distribution in physical, psychological and cognitive co-morbidity among participants | | | | | | | | |
| --- | --- | --- | --- | --- | --- | --- | --- | --- |
| **Variables** | **Total**  **(n = 6057)** | **Physical co-morbidity**  **(n = 4535)** | **Psychological co-morbidity**  **(n = 1971)** | **Cognitive co-morbidity**  **(n = 1800)** | **P-Ps co-morbidity**  **(n = 1585)** | **P-C co-morbidity**  **(n = 1390)** | **Ps-C co-morbidity**  **(n = 711)** | **P-Ps-C co-morbidity**  **(n = 594)** |
| **Sex** |  |  |  |  |  |  |  |  |
| Male | 2187 (36.11) | 1576 (34.75) | 529 (26.84) | 621 (34.50) | 411 (25.93) | 472 (33.96) | 197 (27.71) | 160 (26.94) |
| Female | 3870 (63.89) | 2959 (65.25) | 1442 (73.16) | 1179 (65.50) | 1174 (74.07) | 918 (66.04) | 514 (72.29) | 434 (73.06) |
| **Age** |  |  |  |  |  |  |  |  |
| 65-70 | 2544 (42.00) | 1862 (41.06) | 828 (42.01) | 655 (36.39) | 661 (41.70) | 495 (35.61) | 266 (37.41) | 220 (37.04) |
| 71-80 | 1927 (31.81) | 1474 (32.50) | 658 (33.38) | 557 (30.94) | 531 (33.50) | 445 (32.01) | 236 (33.19) | 201 (33.84) |
| 81-90 | 1080 (17.83) | 830 (18.30) | 329 (16.69) | 375 (20.83) | 265 (16.72) | 296 (21.29) | 135 (18.99) | 109 (18.35) |
| ≥ 91 | 506 (8.35) | 369 (8.14) | 156 (7.91) | 213 (11.83) | 128 (8.08) | 154 (11.08) | 74 (10.41) | 64 (10.77) |
| **Marital status** |  |  |  |  |  |  |  |  |
| Unmarried | 160 (2.64) | 100 (2.21) | 52 (2.64) | 57 (3.17) | 32 (2.02) | 36 (2.59) | 17 (2.39) | 11 (1.85) |
| Married | 4521 (74.64) | 3385 (74.64) | 1461 (74.12) | 1322 (73.44) | 1184 (74.70) | 1038 (74.68) | 540 (75.95) | 455 (76.60) |
| Widowed | 1376 (22.72) | 1050 (23.15) | 458 (23.24) | 421 (23.39) | 369 (23.28) | 316 (22.73) | 154 (21.66) | 128 (21.55) |
| **Education** |  |  |  |  |  |  |  |  |
| Illiteracy | 2696 (44.51) | 2006 (44.23) | 969 (49.16) | 710 (39.44) | 752 (47.44) | 535 (38.49) | 296 (41.63) | 232 (39.06) |
| Primary school | 1927 (31.81) | 1475 (32.52) | 631 (32.01) | 560 (31.11) | 535 (33.75) | 448 (32.23) | 236 (33.19) | 212 (35.69) |
| Junior high school | 1028 (16.97) | 756 (16.67) | 284 (14.41) | 430 (23.89) | 230 (14.51) | 329 (23.67) | 150 (21.10) | 127 (21.38) |
| Senior high school and above | 406 (6.70) | 298 (6.57) | 87 (4.41) | 100 (5.56) | 68 (4.29) | 78 (5.61) | 29 (4.08) | 23 (3.87) |
| **Occupation** |  |  |  |  |  |  |  |  |
| Agriculture | 3775 (62.32) | 2774 (61.17) | 1257 (63.77) | 1150 (63.89) | 998 (62.97) | 866 (62.30) | 468 (65.82) | 389 (65.49) |
| Non-agriculture | 1889 (31.19) | 1462 (32.24) | 616 (31.25) | 545 (30.28) | 505 (31.86) | 438 (31.51) | 203 (28.55) | 170 (28.62) |
| Retirement | 393 (6.49) | 299 (6.59) | 98 (4.97) | 105 (5.83) | 82 (5.17) | 86 (6.19) | 40 (5.63) | 35 (5.89) |
| **Duration of disease** |  |  |  |  |  |  |  |  |
| ≤5 years | 2092 (34.54) | 1520 (33.52) | 579 (29.38) | 590 (32.78) | 455 (28.71) | 449 (32.30) | 192 (27.00) | 159 (26.77) |
| 6-10 years | 1452 (23.97) | 1112 (24.52) | 481 (24.40) | 423 (23.50) | 400 (25.24) | 318 (22.88) | 164 (23.07) | 135 (22.73) |
| 11-15 years | 1347 (22.24) | 1027 (22.65) | 476 (24.15) | 417 (23.17) | 391 (24.67) | 330 (23.74) | 178 (25.04) | 152 (25.59) |
| 16-20 years | 681 (11.24) | 503 (11.09) | 236 (11.97) | 209 (11.61) | 179 (11.29) | 170 (12.23) | 92 (12.94) | 78 (13.13) |
| ≥21 years | 485 (8.01) | 373 (8.22) | 199 (10.10) | 161 (8.94) | 160 (10.09) | 123 (8.85) | 85 (11.95) | 70 (11.78) |
| **Complication** |  |  |  |  |  |  |  |  |
| Yes | 3250 (53.66) | 2561 (56.47) | 1212 (61.49) | 1012 (56.22) | 999 (63.03) | 822 (59.14) | 435 (61.18) | 369 (62.12) |
| No | 2807 (46.34) | 1974 (43.53) | 759 (38.51) | 788 (43.78) | 586 (36.97) | 568 (40.86) | 276 (38.82) | 225 (37.88) |
| **BMI** |  |  |  |  |  |  |  |  |
| 18.5-23.9 | 2190 | 1521 (33.54) | 719 (36.48) | 719 (36.48) | 547 (34.51) | 521 (37.48) | 286 (40.23) | 229 (38.55) |
| >23.9 | 3664 | 2892 (63.77) | 1184 (60.07) | 1184 (60.07) | 993 (62.65) | 826 (59.42) | 400 (56.26) | 348 (58.59) |
| <18.5 | 203 | 122 (2.69) | 68 (3.45) | 68 (3.45) | 45 (2.84) | 43 (3.09) | 25 (3.52) | 17 (2.86) |
| **Take medicine** |  |  |  |  |  |  |  |  |
| No | 477 (7.88) | 335 (7.39) | 124 (6.29) | 136 (7.56) | 101 (6.37) | 94 (6.76) | 42 (5.91) | 35 (5.89) |
| Yes | 5580 (92.12) | 4200 (92.61) | 1847 (93.71) | 1664 (92.44) | 1484 (93.63) | 1296 (93.24) | 669 (94.09) | 559 (94.11) |
| FPG (mean) | 6.53±3.01 | 6.53±3.08 | 6.51±2.91 | 6.43±2.80 | 6.51±2.98 | 6.46±2.83 | 6.43±2.71 | 6.44±2.80 |
| **Physical activity** |  |  |  |  |  |  |  |  |
| No exercise | 1731 (28.58) | 1358 (29.94) | 618 (31.35) | 687 (38.17) | 510 (32.18) | 547 (39.35) | 283 (39.80) | 237 (39.90) |
| Exercise | 4326 (71.42) | 3177 (70.06) | 1353 (68.65) | 1113 (61.83) | 1075 (67.82) | 843 (60.65) | 428 (60.20) | 357 (60.10) |
| **Diet** |  |  |  |  |  |  |  |  |
| Unhealthy | 4856 (80.17) | 3628 (80.00) | 1650 (83.71) | 1475 (81.94) | 1322 (83.41) | 1139 (81.94) | 600 (84.39) | 502 (84.51) |
| Healthy | 1201 (19.83) | 907 (20.00) | 321 (16.29) | 325 (18.06) | 263 (16.59) | 251 (18.06) | 111 (15.61) | 92 (15.49) |
| **Smoking** |  |  |  |  |  |  |  |  |
| Current | 1248 (20.60) | 894 (19.71) | 322 (16.34) | 339 (18.83) | 247 (15.58) | 253 (18.20) | 116 (16.32) | 92 (15.49) |
| Never | 4809 (79.40) | 3641 (80.29) | 1649 (83.66) | 1461 (81.17) | 1338 (84.42) | 1137 (81.80) | 595 (83.68) | 502 (84.51) |
| **Sleep** |  |  |  |  |  |  |  |  |
| <7 hour/>8 hour | 2333 (38.52) | 1780 (39.25) | 901 (45.71) | 765 (42.50) | 737 (46.50) | 614 (44.17) | 340 (47.82) | 295 (49.66) |
| 7-8 hours | 3724 (61.48) | 2755 (60.75) | 1070 (54.29) | 1035 (57.50) | 848 (53.50) | 776 (55.83) | 371 (52.18) | 299 (50.34) |
| **Social participation** |  |  |  |  |  |  |  |  |
| No | 2880 (47.55) | 2196 (48.42) | 1038 (52.66) | 1002 (55.67) | 844 (53.25) | 789 (56.76) | 432 (60.76) | 359 (60.44) |
| Yes | 3177 (52.45) | 2339 (51.58) | 933 (47.34) | 798 (44.33) | 741 (46.75) | 601 (43.24) | 279 (39.24) | 235 (39.56) |
| Note: P-Ps co-morbidity for physical-psychological co-morbidity; P-C co-morbidity for physical-cognitive co-morbidity; Ps-C co-morbidity for psychological-cognitive co-morbidity; P-Ps-C co-morbidity for physical-psychological-cognitive co-morbidity. | | | | | | | | |

| **Table.S4.** Prevalence rate of individual physical co-morbidity, psychological co-morbidity and cognitive co-morbidity of the participants | | | | | | | |
| --- | --- | --- | --- | --- | --- | --- | --- |
| **Variables** | **Total (N=6075)** | **Physical**  **co-morbidity** | ***P*** | **Psychological**  **co-morbidity** | ***P*** | **Cognitive**  **co-morbidity** | ***P*** |
| **Sex** |  |  | <0.001 |  | <0.001 |  | 0.09 |
| Male | 2187 (36.11) | 72.06(70.14-73.90) |  | 24.19(22.44-26.03) |  | 28.40(26.54-30.32) |  |
| Female | 3870 (63.89) | 76.46(75.10-77.77) |  | 37.26(35.75-38.80) |  | 30.47(29.03-31.93) |  |
| **Age** |  |  | 0.021 |  | 0.167 |  | <0.001 |
| 65-70 | 2544 (42.00) | 73.19(71.44-74.88) |  | 32.55(30.75-34.39) |  | 25.75(24.08-27.48) |  |
| 71-80 | 1927 (31.81) | 76.49(74.55-78.33) |  | 34.15(32.06-36.29) |  | 28.91(26.92-30.97) |  |
| 81-90 | 1080 (17.83) | 76.85(74.24-79.27) |  | 30.46(27.79-33.28) |  | 34.72(31.94-37.61) |  |
| ≥ 91 | 506 (8.35) | 72.92(68.88-76.62) |  | 30.83(26.96-34.99) |  | 42.09(37.86-46.45) |  |
| **Marital status** |  |  | <0.001 |  | 0.798 |  | 0.159 |
| Unmarried | 160 (2.64) | 62.50(54.75-69.66) |  | 32.50(25.70-40.13) |  | 35.63(28.59-43.34) |  |
| Married | 4521 (74.64) | 74.87(73.59-76.12) |  | 32.32(30.97-33.69) |  | 29.24(27.93-30.58) |  |
| Widowed | 1376 (22.72) | 76.31(73.99-78.48) |  | 33.28(30.84-35.82) |  | 30.60(28.22-33.08) |  |
| **Education** |  |  | 0.203 |  | <0.001 |  | <0.001 |
| Illiteracy | 2696 (44.51) | 74.41(72.72-76.02) |  | 35.94(34.15-37.77) |  | 26.34(24.71-28.03) |  |
| Primary school | 1927 (31.81) | 76.54(74.60-78.38) |  | 32.75(30.68-34.87) |  | 29.06(27.08-31.13) |  |
| Junior high school | 1028 (16.97) | 73.54(70.76-76.15) |  | 27.63(24.98-30.44) |  | 41.83(38.85-44.87) |  |
| Senior high school and above | 406 (6.70) | 73.40(68.89-77.47) |  | 21.43(17.71-25.69) |  | 24.63(20.68-29.06) |  |
| **Occupation** |  |  | 0.005 |  | 0.003 |  | 0.185 |
| Agriculture | 3775 (62.32) | 73.48(72.05-74.87) |  | 33.30(31.81-34.82) |  | 30.46(29.02-31.95) |  |
| Non-agriculture | 1889 (31.19) | 77.35(75.41-79.19) |  | 32.59(30.52-34.74) |  | 28.84(26.84-30.92) |  |
| Retirement | 393 (6.49) | 76.28(71.81-80.23) |  | 25.00(20.96-29.53) |  | 26.79(22.63-31.39) |  |
| **Duration of disease** |  |  | 0.033 |  | <0.001 |  | 0.156 |
| ≤5 years | 2092 (34.54) | 72.66(70.71-74.53) |  | 27.68(25.80-29.63) |  | 28.20(26.31-30.17) |  |
| 6-10 years | 1452 (23.97) | 76.58(74.34-78.69) |  | 33.13(30.75-35.59) |  | 29.13(26.85-31.52) |  |
| 11-15 years | 1347 (22.24) | 76.24(73.90-78.44) |  | 35.34(32.83-37.93) |  | 30.96(28.54-33.48) |  |
| 16-20 years | 681 (11.24) | 73.86(70.43-77.03) |  | 34.65(31.17-38.31) |  | 30.69(27.34-34.26) |  |
| ≥21 years | 485 (8.01) | 76.91(72.95-80.44) |  | 41.03(36.73-45.47) |  | 33.20(29.14-37.51) |  |
| **Complication** |  |  | <0.001 |  | <0.001 |  | 0.009 |
| Yes | 3250 (53.66) | 78.80(77.36-80.17) |  | 37.29(35.65-38.97) |  | 31.14(29.57-32.75) |  |
| No | 2807 (46.34) | 70.32(68.61-71.99) |  | 27.04(25.43-28.71) |  | 28.07(26.44-29.76) |  |
| **BMI** |  |  | <0.001 |  | 0.881 |  | <0.001 |
| 18.5-23.9 | 2190 | 78.93(77.58-80.22) |  | 32.31(30.82-33.85) |  | 27.67(26.25-29.15) |  |
| >23.9 | 3664 | 60.10(53.21-66.61) |  | 33.50(27.34-40.27) |  | 34.98(28.73-41.78) |  |
| <18.5 | 203 | 69.45(67.49-71.35) |  | 32.83(30.89-34.83) |  | 32.65(30.72-34.64) |  |
| **Take medicine** |  |  | 0.015 |  | <0.001 |  | 0.548 |
| No | 477 (7.88) | 70.23(65.97-74.17) |  | 26.00(22.26-30.12) |  | 28.51(24.64-32.73) |  |
| Yes | 5580 (92.12) | 75.27(74.12-76.38) |  | 33.10(31.88-34.35) |  | 29.82(28.63-31.04) |  |
| **Physical activity** |  |  | <0.001 |  | <0.001 |  | <0.001 |
| No exercise | 1731 (28.58) | 78.45(76.45-80.33) |  | 35.70(33.48-37.99) |  | 39.69(37.41-42.01) |  |
| Exercise | 4326 (71.42) | 73.44(72.10-74.74) |  | 31.28(29.91-32.67) |  | 25.73(24.45-27.05) |  |
| **Diet** |  |  | 0.563 |  | <0.001 |  | 0.024 |
| Unhealthy | 4856 (80.17) | 74.71(73.47-75.91) |  | 33.98(32.66-35.32) |  | 30.37(29.10-31.68) |  |
| Healthy | 1201 (19.83) | 75.52(73.01-77.87) |  | 26.73(24.30-29.30) |  | 27.06(24.62-29.65) |  |
| **Smoking** |  |  | 0.003 |  | <0.001 |  | 0.027 |
| Current | 1248 (20.60) | 70.11(66.61-73.40) |  | 24.71(21.65-28.06) |  | 21.12(18.25-24.31) |  |
| Never | 4809 (79.40) | 75.49(74.32-76.62) |  | 33.56(32.30-34.83) |  | 30.83(29.61-32.08) |  |
| **Sleep** |  |  | 0.043 |  | <0.001 |  | <0.001 |
| <7 hour/>8 hour | 2333 (38.52) | 76.30(74.53-77.98) |  | 38.62(36.66-40.61) |  | 32.79(30.91-34.72) |  |
| 7-8 hours | 3724 (61.48) | 73.98(72.55-75.36) |  | 28.73(27.30-30.21) |  | 27.79(26.38-29.25) |  |
| **Social participation** |  |  | 0.019 |  | <0.001 |  | <0.001 |
| No | 2880 (47.55) | 76.25(74.66-77.77) |  | 36.04(34.31-37.81) |  | 34.79(33.07-36.55) |  |
| Yes | 3177 (52.45) | 73.62(72.06-75.13) |  | 29.37(27.81-30.98) |  | 25.12(23.64-26.66) |  |
| *P* for Chi-square test | | | | | | | |

| **Table.S5.** Prevalence rate of four combination among participants | | | | | | | | | |
| --- | --- | --- | --- | --- | --- | --- | --- | --- | --- |
| **Variables** | **Total**  **(n = 6057)** | **P-Ps**  **co-morbidity** | ***P*** | **P-C**  **co-morbidity** | ***P*** | **Ps-C co-morbidity** | ***P*** | **P-Ps-C co-morbidity** | ***P*** |
| **Sex** |  |  | <0.001 |  | 0.057 |  | <0.001 |  | <0.001 |
| Male | 2187 (36.11) | 21.58(19.91-23.36) |  | 21.58(19.91-23.36) |  | 9.01(7.88-10.28) |  | 7.32(6.30-8.49) |  |
| Female | 3870 (63.89) | 23.72(22.41-25.09) |  | 23.72(22.41-25.09) |  | 13.28(12.25-14.39) |  | 11.21(10.26-12.25) |  |
| **Age** |  |  | 0.302 |  | <0.001 |  | 0.027 |  | 0.024 |
| 65-70 | 2544 (42.00) | 19.46(17.96-21.04) |  | 19.46(17.96-21.04) |  | 10.46(9.33-11.71) |  | 8.65(7.62-9.80) |  |
| 71-80 | 1927 (31.81) | 23.09(21.26-25.03) |  | 23.09(21.26-25.03) |  | 12.25(10.86-13.79) |  | 10.43(9.14-11.88) |  |
| 81-90 | 1080 (17.83) | 27.41(24.83-30.15) |  | 27.41(24.83-30.15) |  | 12.50(10.66-14.61) |  | 10.09(8.43-12.04) |  |
| ≥ 91 | 506 (8.35) | 30.43(26.58-34.59) |  | 30.43(26.58-34.59) |  | 14.62(11.81-17.98) |  | 12.65(10.02-15.84) |  |
| **Marital status** |  |  | 0.178 |  | 0.991 |  | 0.679 |  | 0.318 |
| Unmarried | 160 (2.64) | 22.50(16.69-29.62) |  | 22.50(16.69-29.62) |  | 10.63(6.71-16.43) |  | 6.88(3.85-11.99) |  |
| Married | 4521 (74.64) | 22.96(21.76-24.21) |  | 22.96(21.76-24.21) |  | 11.94(11.03-12.92) |  | 10.06(9.22-10.98) |  |
| Widowed | 1376 (22.72) | 22.97(20.82-25.26) |  | 22.97(20.82-25.26) |  | 11.19(9.63-12.97) |  | 9.30(7.88-10.96) |  |
| **Education** |  |  | <0.001 |  | <0.001 |  | <0.001 |  | <0.001 |
| Illiteracy | 2696 (44.51) | 19.84(18.38-21.39) |  | 19.84(18.38-21.39) |  | 10.98(9.85-12.22) |  | 8.61(7.60-9.72) |  |
| Primary school | 1927 (31.81) | 23.25(21.42-25.19) |  | 23.25(21.42-25.19) |  | 12.25(10.86-13.79) |  | 11.00(9.68-12.48) |  |
| Junior high school | 1028 (16.97) | 32.00(29.22-34.92) |  | 32.00(29.22-34.92) |  | 14.59(12.56-16.88) |  | 12.35(10.48-14.51) |  |
| Senior high school and above | 406 (6.70) | 19.21(15.67-23.34) |  | 19.21(15.67-23.34) |  | 7.14(5.01-10.09) |  | 5.67(3.79-8.38) |  |
| **Occupation** |  |  | 0.046 |  | 0.855 |  | 0.117 |  | 0.245 |
| Agriculture | 3775 (62.32) | 22.94(21.63-24.31) |  | 22.94(21.63-24.31) |  | 12.40(11.38-13.49) |  | 10.30(9.37-11.32) |  |
| Non-agriculture | 1889 (31.19) | 23.17(21.33-25.13) |  | 23.17(21.33-25.13) |  | 10.74(9.42-12.22) |  | 8.99(7.79-10.37) |  |
| Retirement | 393 (6.49) | 21.94(18.12-26.31) |  | 21.94(18.12-26.31) |  | 10.20(7.57-13.61) |  | 8.93(6.48-12.18) |  |
| **Duration of disease** |  |  | <0.001 |  | 0.075 |  | <0.001 |  | <0.001 |
| ≤5 years | 2092 (34.54) | 21.46(19.76-23.27) |  | 21.46(19.76-23.27) |  | 9.18(8.01-10.49) |  | 7.60(6.54-8.82) |  |
| 6-10 years | 1452 (23.97) | 21.90(19.85-24.10) |  | 21.90(19.85-24.10) |  | 11.29(9.77-13.03) |  | 9.30(7.91-10.90) |  |
| 11-15 years | 1347 (22.24) | 24.50(22.27-26.87) |  | 24.50(22.27-26.87) |  | 13.21(11.51-15.13) |  | 11.28(9.70-13.09) |  |
| 16-20 years | 681 (11.24) | 24.96(21.85-28.35) |  | 24.96(21.85-28.35) |  | 13.51(11.14-16.29) |  | 11.45(9.27-14.07) |  |
| ≥21 years | 485 (8.01) | 25.36(21.69-29.42) |  | 25.36(21.69-29.42) |  | 17.53(14.39-21.17) |  | 14.43(11.58-17.85) |  |
| **Complication** |  |  | <0.001 |  | <0.001 |  | <0.001 |  | <0.001 |
| Yes | 3250 (53.66) | 25.29(23.83-26.82) |  | 25.29(23.83-26.82) |  | 13.38(12.26-14.60) |  | 11.35(10.31-12.49) |  |
| No | 2807 (46.34) | 20.24(18.79-21.76) |  | 20.24(18.79-21.76) |  | 9.83(8.78-10.99) |  | 8.02(7.07-9.08) |  |
| **BMI** |  |  | 0.085 |  | 0.455 |  | 0.046 |  | 0.384 |
| 18.5-23.9 | 2190 | 22.54(21.22-23.93) |  | 22.54(21.22-23.93) |  | 10.92(9.95-11.97) |  | 9.50(8.59-10.49) |  |
| >23.9 | 3664 | 21.18(16.10-27.34) |  | 21.18(16.10-27.34) |  | 12.32(8.46-17.59) |  | 8.37(5.27-13.06) |  |
| <18.5 | 203 | 23.79(22.05-25.62) |  | 23.79(22.05-25.62) |  | 13.06(11.71-14.54) |  | 10.46(9.24-11.81) |  |
| **Take medicine** |  |  | 0.01 |  | 0.079 |  | 0.038 |  | 0.059 |
| No | 477 (7.88) | 19.71(16.38-23.52) |  | 19.71(16.38-23.52) |  | 8.81(6.57-11.70) |  | 7.34(5.31-10.05) |  |
| Yes | 5580 (92.12) | 23.23(22.14-24.35) |  | 23.23(22.14-24.35) |  | 11.99(11.16-12.87) |  | 10.02(9.26-10.83) |  |
| **Physical activity** |  |  | <0.001 |  | <0.001 |  | <0.001 |  | <0.001 |
| No exercise | 1731 (28.58) | 31.60(29.45-33.83) |  | 31.60(29.45-33.83) |  | 16.35(14.68-18.17) |  | 13.69(12.15-15.39) |  |
| Exercise | 4326 (71.42) | 19.49(18.33-20.69) |  | 19.49(18.33-20.69) |  | 9.89(9.04-10.82) |  | 8.25(7.47-9.11) |  |
| **Diet** |  |  | <0.001 |  | 0.059 |  | <0.001 |  | 0.005 |
| Unhealthy | 4856 (80.17) | 23.46(22.28-24.67) |  | 23.46(22.28-24.67) |  | 12.36(11.46-13.31) |  | 10.34(9.51-11.23) |  |
| Healthy | 1201 (19.83) | 20.90(18.69-23.29) |  | 20.90(18.69-23.29) |  | 9.24(7.73-11.02) |  | 7.66(6.28-9.31) |  |
| **Smoking** |  |  | <0.001 |  | 0.012 |  | 0.003 |  | 0.001 |
| Current | 1248 (20.60) | 15.80(13.28-18.71) |  | 15.80(13.28-18.71) |  | 6.90(5.24-9.03) |  | 5.17(3.75-7.09) |  |
| Never | 4809 (79.40) | 23.88(22.75-25.04) |  | 23.88(22.75-25.04) |  | 12.37(11.51-13.28) |  | 10.41(9.62-11.25) |  |
| **Sleep** |  |  | <0.001 |  | <0.001 |  | <0.001 |  | <0.001 |
| <7 hour/>8 hour | 2333 (38.52) | 26.32(24.57-28.14) |  | 26.32(24.57-28.14) |  | 14.57(13.20-16.06) |  | 12.64(11.36-14.06) |  |
| 7-8 hours | 3724 (61.48) | 20.84(19.56-22.17) |  | 20.84(19.56-22.17) |  | 9.96(9.04-10.97) |  | 8.03(7.20-8.95) |  |
| **Social participation** |  |  | <0.001 |  | <0.001 |  | <0.001 |  | <0.001 |
| No | 2880 (47.55) | 27.40(25.80-29.05) |  | 27.40(25.80-29.05) |  | 15.00(13.74-16.35) |  | 12.47(11.31-13.72) |  |
| Yes | 3177 (52.45) | 18.92(17.59-20.32) |  | 18.92(17.59-20.32) |  | 8.78(7.85-9.82) |  | 7.40(6.54-8.36) |  |
| **Note:** P-Ps co-morbidity for physical-psychological co-morbidity; P-C co-morbidity for physical-cognitive co-morbidity; Ps-C co-morbidity for psychological-cognitive co-morbidity; P-Ps-C co-morbidity for physical-psychological-cognitive co-morbidity. *p*: Chi-square test; | | | | | | | | | |

**
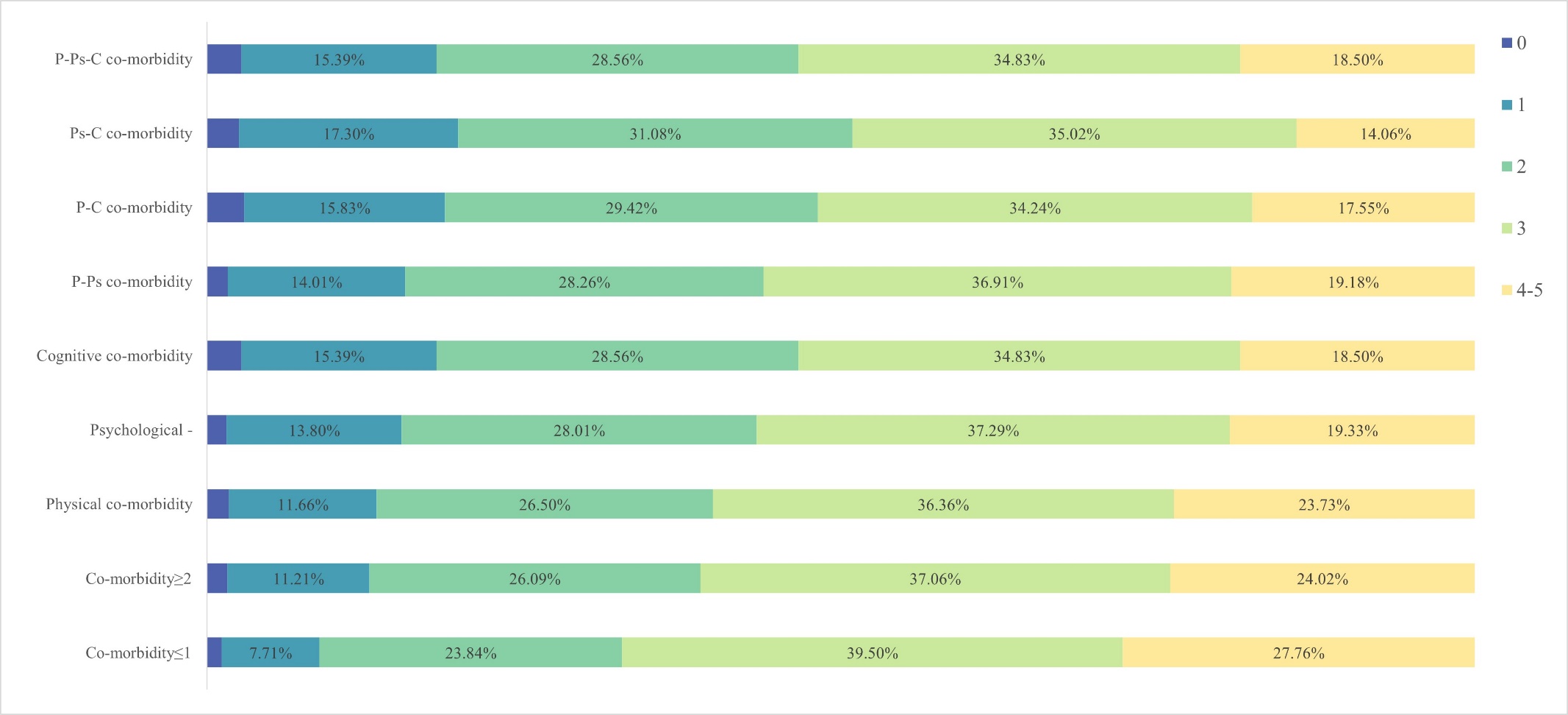
**

**Fig.S1.** Stacking plot of lifestyle scores in physical, psychological and cognitive co-morbidity

**Note:** P-Ps co-morbidity for physical-psychological co-morbidity; P-C co-morbidity for physical-cognitive co-morbidity; Ps-C co-morbidity for psychological-cognitive co-morbidity; P-Ps-C co-morbidity for physical-psychological-cognitive co-morbidity.

| **Table.S6.** Basic characteristics of lifestyle scores in among older adults with diabetes | | | | | | | | |
| --- | --- | --- | --- | --- | --- | --- | --- | --- |
| **Variables** | **Lifestyles Scores** | | | | | | | ***P*** |
|  | Total  (n = 6057) | | 0  (n = 98) | 1  (n = 679) | 2  (n = 1580) | 3  (n = 2245) | 4-5  (n = 1455) |  |
|  |  | |  |  |  |  |  | <0.001 |
| **Sex** |  | |  |  |  |  |  |  |
| Male | 2187 (36.11) | | 96 (97.96) | 385 (56.70) | 692 (43.80) | 716 (31.89) | 298 (20.48) |  |
| Female | 3870 (63.89) | | 2 (2.04) | 294 (43.30) | 888 (56.20) | 1529 (68.11) | 1157 (79.52) |  |
| **Age** |  | |  |  |  |  |  | <0.001 |
| 65-70 | 2544 (42.00) | | 28 (28.57) | 222 (32.70) | 636 (40.25) | 971 (43.25) | 687 (47.22) |  |
| 71-80 | 1927 (31.81) | | 31 (31.63) | 227 (33.43) | 480 (30.38) | 717 (31.94) | 472 (32.44) |  |
| 81-90 | 1080 (17.83) | | 22 (22.45) | 142 (20.91) | 308 (19.49) | 395 (17.59) | 213 (14.64) |  |
| ≥91 | 506 (8.35) | | 17 (17.35) | 88 (12.96) | 156 (9.87) | 162 (7.22) | 83 (5.70) |  |
| **Marital status** |  | |  |  |  |  |  | 0.049 |
| Unmarried | 160 (2.64) | | 5 (5.10) | 18 (2.65) | 39 (2.47) | 70 (3.12) | 28 (1.92) |  |
| Married | 4521 (74.64) | | 78 (79.59) | 509 (74.96) | 1163 (73.61) | 1648 (73.41) | 1123 (77.18) |  |
| Widowed | 1376 (22.72) | | 15 (15.31) | 152 (22.39) | 378 (23.92) | 527 (23.47) | 304 (20.89) |  |
| **Education** |  | |  |  |  |  |  | 0.002 |
| Illiteracy | 2696 (44.51) | | 24 (24.49) | 279 (41.09) | 690 (43.67) | 1022 (45.52) | 681 (46.80) |  |
| Primary school | 1927 (31.81) | | 42 (42.86) | 221 (32.55) | 523 (33.10) | 693 (30.87) | 448 (30.79) |  |
| Junior high school | 1028 (16.97) | | 26 (26.53) | 134 (19.73) | 264 (16.71) | 383 (17.06) | 221 (15.19) |  |
| Senior high school and above | 406 (6.70) | | 6 (6.12) | 45 (6.63) | 103 (6.52) | 147 (6.55) | 105 (7.22) |  |
| **Occupation** |  | |  |  |  |  |  | 0.280 |
| Agriculture | 3775 (62.32) | | 62 (63.27) | 423 (62.30) | 996 (63.04) | 1426 (63.52) | 868 (59.66) |  |
| Non-agriculture | 1890 (31.20) | | 26 (26.53) | 208 (30.63) | 487 (30.82) | 684 (30.47) | 485 (33.33) |  |
| Retirement | 392 (6.47) | | 10 (10.20) | 48 (7.07) | 97 (6.14) | 135 (6.01) | 102 (7.01) |  |
| **Duration of disease** |  | |  |  |  |  |  | 0.320 |
| ≤5 years | 2092 (34.54) | | 35 (35.71) | 230 (33.87) | 549 (34.75) | 781 (34.79) | 497 (34.16) |  |
| 6-10 years | 1452 (23.97) | | 23 (23.47) | 165 (24.30) | 351 (22.22) | 546 (24.32) | 367 (25.22) |  |
| 11-15 years | 1347 (22.24) | | 19 (19.39) | 146 (21.50) | 370 (23.42) | 501 (22.32) | 311 (21.37) |  |
| 16-20 years | 681 (11.24) | | 16 (16.33) | 65 (9.57) | 183 (11.58) | 251 (11.18) | 166 (11.41) |  |
| ≥20 years | 485 (8.01) | | 5 (5.10) | 73 (10.75) | 127 (8.04) | 166 (7.39) | 114 (7.84) |  |
| **Complication** |  | |  |  |  |  |  | 0.032 |
| No | 3250 (53.66) | | 55 (56.12) | 383 (56.41) | 886 (56.08) | 1156 (51.49) | 770 (52.92) |  |
| Yes | 2807 (46.34) | | 43 (43.88) | 296 (43.59) | 694 (43.92) | 1089 (48.51) | 685 (47.08) |  |
| **BMI** |  | |  |  |  |  |  | 0.748 |
| 18.5-23.9 | 2190 (36.16) | | 32 (32.65) | 245 (36.08) | 598 (37.85) | 804 (35.81) | 511 (35.12) |  |
| <18.5 | 3664 (60.49) | | 64 (65.31) | 407 (59.94) | 931 (58.92) | 1364 (60.76) | 898 (61.72) |  |
| >23.9 | 203 (3.35) | | 2 (2.04) | 27 (3.98) | 51 (3.23) | 77 (3.43) | 46 (3.16) |  |
| **Take medicine** |  | |  |  |  |  |  | 0.349 |
| No | 477 (7.88) | | 9 (9.18) | 42 (6.19) | 121 (7.66) | 192 (8.55) | 113 (7.77) |  |
| Yes | 5580 (92.12) | | 89 (90.82) | 637 (93.81) | 1459 (92.34) | 2053 (91.45) | 1342 (92.23) |  |
| *Note: P* for Chi-square test | |  |  |  |  |  |  |  |

| **Table.S7.** The association of lifestyle category with co-morbidity | | | | | |
| --- | --- | --- | --- | --- | --- |
| **Co-morbidity Status** | **N*** | Crude OR | *P* | Adjusted OR† | *P* |
| **Co-morbidity≤1** |  |  |  |  |  |
| Lifestyle scores<3 | 961 (35.38) | Reference |  | Reference |  |
| Lifestyle scores≥3 | 1755 (64.62) | 0.76(0.69-0.85) | <0.001 | 0.73(0.65-0.81) | <0.001 |
| **Co-morbidity≥2** |  |  |  |  |  |
| Lifestyle scores<3 | 1120 (44.84) | Reference |  | Reference |  |
| Lifestyle scores≥3 | 1378 (55.16) | 0.66(0.59-0.73) | <0.001 | 0.59(0.53-0.66) | 0.001 |
| **Different subgroups** |  |  |  |  |  |
| **Physical co-morbidity** |  |  |  |  |  |
| Lifestyle scores<3 | 1810 (39.91) | Reference |  | Reference |  |
| Lifestyle scores≥3 | 2725 (60.09) | 0.84(0.75-0.95) | 0.006 | 0.81(0.71-0.92) | <0.001 |
| **Psychological co-morbidity** |  |  |  |  |  |
| Lifestyle scores<3 | 855 (43.38) |  |  |  |  |
| Lifestyle scores≥3 | 1116 (56.62) | 0.76(0.68-0.85) | <0.001 | 0.66(0.59-0.74) | <0.001 |
| **Cognitive co-morbidity** |  |  |  |  |  |
| Lifestyle scores<3 | 840 (46.67) | Reference |  | Reference |  |
| Lifestyle scores≥3 | 960 (53.33) | 0.63(0.57-0.71) | <0.001 | 0.61(0.55-0.69) | <0.001 |
| **P-Ps co-morbidity** |  |  |  |  |  |
| Lifestyle scores<3 | 696 (43.91) | Reference |  | Reference |  |
| Lifestyle scores≥3 | 889 (56.09) | 0.75(0.67-0.85) | <0.001 | 0.66(0.58-0.74) | <0.001 |
| **P-C co-morbidity** |  |  |  |  |  |
| Lifestyle scores<3 | 670 (48.20) | Reference |  | Reference |  |
| Lifestyle scores≥3 | 720 (51.80) | 0.61(0.54-0.69) | <0.001 | 0.59(0.52-0.67) | <0.001 |
| **Ps-C co-morbidity** |  |  |  |  |  |
| Lifestyle scores<3 | 362 (50.91) | Reference |  | Reference |  |
| Lifestyle scores≥3 | 349 (49.09) | 0.57(0.49-0.67) | <0.001 | 0.52(0.44-0.61) | <0.001 |
| **P-Ps-C co-morbidity** |  |  |  |  |  |
| Lifestyle scores<3 | 304 (51.18) | Reference |  | Reference |  |
| Lifestyle scores≥3 | 290 (48.82) | 0.57(0.48-0.68) | <0.001 | 0.51(0.43-0.61) | <0.001 |
| **Note:** P-Ps co-morbidity for physical-psychological co-morbidity; P-C co-morbidity for physical-cognitive co-morbidity; Ps-C co-morbidity for psychological-cognitive co-morbidity; P-Ps-C co-morbidity for physical-psychological-cognitive co-morbidity. Crude OR refer to no adjustments Adjusted OR incorporated sex, age, education, marital status, occupation, disease duration, complication, medication use, and BMI. | | | | | |

| **Table.S8.** Balance test of PSM for no co-morbidity and co-morbidity samples | | | | | | | |
| --- | --- | --- | --- | --- | --- | --- | --- |
| **Variable** | **Unmatched** | **Mean** | | **%bias** | **%reduct** | **t-test** | |
|  | Matched | Treated Control | |  | \|bias\| | t | p>\| t \| |
| Age | U | 1.82 | 1.94 | -12.3 |  | -3.28 | 0.001 |
|  | M | 1.82 | 1.81 | 1.1 | 90.9 | 0.23 | 0.816 |
| Sex | U | 1.55 | 1.65 | -21.7 |  | -5.94 | 0.000 |
|  | M | 1.55 | 1.56 | -1.5 | 93.3 | -0.29 | 0.765 |
| Marital status | U | 2.16 | 2.21 | -11 |  | -2.91 | 0.004 |
|  | M | 2.16 | 2.15 | 2.4 | 78.6 | 0.5 | 0.614 |
| Duration of disease | U | 2.21 | 2.36 | -12.3 |  | -3.28 | 0.001 |
|  | M | 2.21 | 2.22 | -0.8 | 93.1 | -0.17 | 0.861 |
| Complication | U | 0.58 | 0.44 | 27.9 |  | 7.5 | 0.000 |
|  | M | 0.58 | 0.58 | 0.7 | 97.4 | 0.15 | 0.882 |
| Take medicine | U | 0.90 | 0.92 | -8.6 |  | -2.43 | 0.015 |
|  | M | 0.90 | 0.91 | -3.4 | 60.9 | -0.66 | 0.506 |
| BMI | U | 0.62 | 0.68 | -11.4 |  | -3.15 | 0.002 |
|  | M | 0.62 | 0.60 | 3.9 | 65.9 | 0.79 | 0.431 |

| 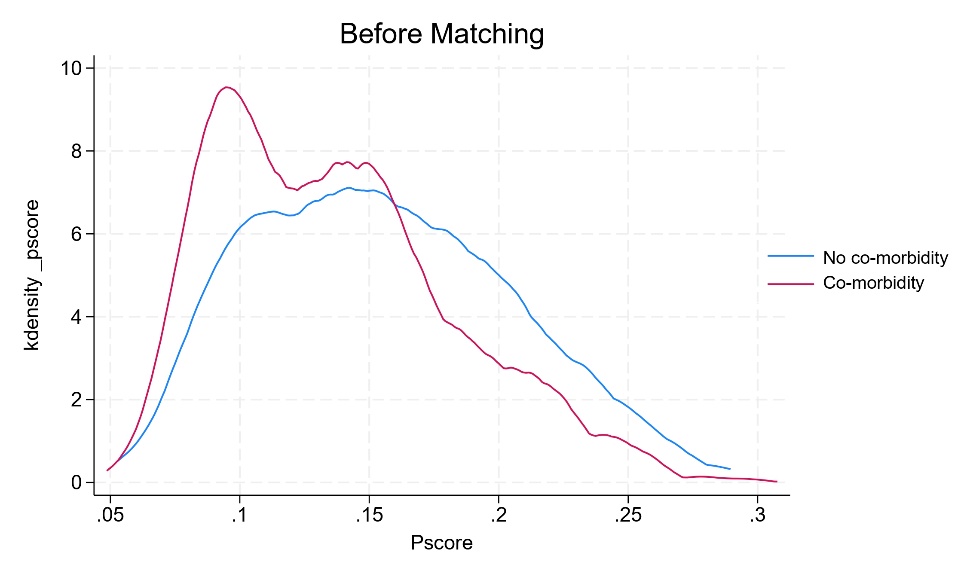 | 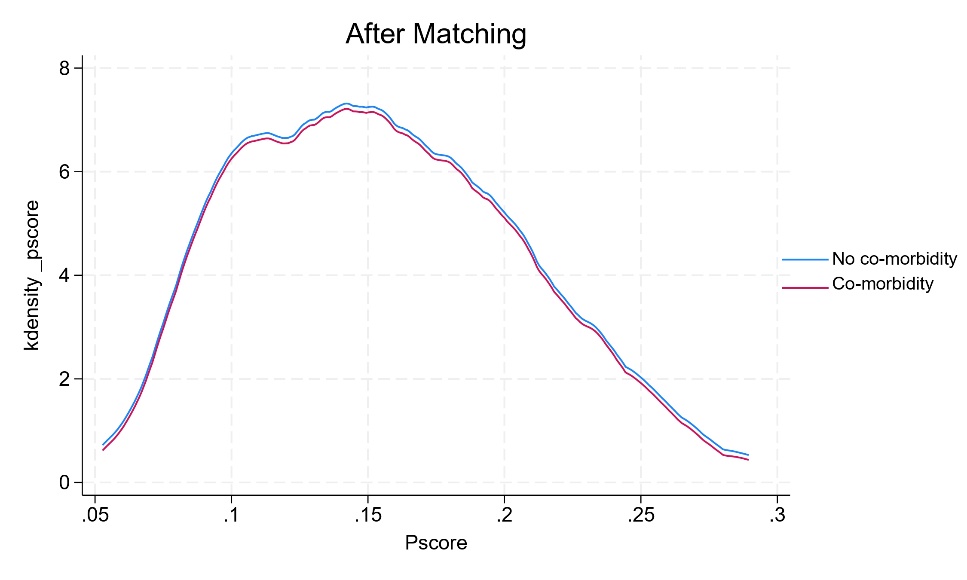 |
| --- | --- |
| **Fig.S2.** Common support domain of PSM for no co-morbidity and co-morbidity groups | |

| **Table.S9.** Differences in lifestyle among no co-morbidity and co-morbidity after PSM | | | | |
| --- | --- | --- | --- | --- |
| **Different Lifestyles** | **Total (%)** | **No co-morbidity** | **Co-morbidity** | ***p*** |
| **Population** | 1686 | 843 | 843 |  |
| **Physical activity** |  |  |  | <0.001 |
| No exercise | 423 (25.09) | 171 (20.28) | 252 (29.89) |  |
| Exercise | 1263 (74.91) | 672 (79.72) | 591 (70.11) |  |
| **Sleep** |  |  |  | <0.001 |
| <7 hour/>8 hour | 613 (36.36) | 283 (33.57) | 330 (39.15) |  |
| 7-8 hours | 1073 (63.64) | 560 (66.43) | 513 (60.85) |  |
| **Social participation** |  |  |  | <0.001 |
| No | 779 (46.20) | 350 (41.52) | 429 (50.89) |  |
| Yes | 907 (53.80) | 493 (58.48) | 414 (49.11) |  |
| **Lifestyle category** |  |  |  | <0.001 |
| Lifestyle scores<3 | 628 (37.25) | 276 (32.74) | 352 (41.76) |  |
| Lifestyle scores≥3 | 1058 (62.75) | 567 (67.26) | 491 (58.24) |  |

| **Table.S10.** Balance test of PSM for one co-morbidity and two or more co-morbidity samples | | | | | | | |
| --- | --- | --- | --- | --- | --- | --- | --- |
| **Variable** | Unmatched | **Mean** | | **%bias** | **%reduct** | **t-test** | |
|  | Matched | Treated Control | |  | \|bias\| | t | p>\| t \| |
| Age | U | 1.98 | 1.89 | 9.8 |  | 3.76 | 0.000 |
|  | M | 1.94 | 1.95 | -1.2 | 87.8 | -0.42 | 0.672 |
| Sex | U | 1.70 | 1.60 | 20.3 |  | 7.75 | 0.000 |
|  | M | 1.69 | 1.69 | -0.6 | 97 | -0.22 | 0.828 |
| Education | U | 1.84 | 1.87 | -3.9 |  | -1.49 | 0.137 |
|  | M | 1.83 | 1.82 | 1.2 | 70.3 | 0.41 | 0.682 |
| Occupation | U | 1.43 | 1.45 | -2.3 |  | -0.89 | 0.376 |
|  | M | 1.44 | 1.43 | 2 | 15.6 | 0.69 | 0.489 |
| Duration of disease | U | 2.43 | 2.28 | 11.6 |  | 4.44 | 0.000 |
|  | M | 2.39 | 2.38 | 0.4 | 96.6 | 0.14 | 0.892 |
| Complication | U | 0.39 | 0.51 | -24.5 |  | -9.36 | 0.000 |
|  | M | 0.40 | 0.41 | -0.3 | 99 | -0.09 | 0.930 |
| Take medicine | U | 0.93 | 0.91 | 7.6 |  | 2.88 | 0.004 |
|  | M | 0.93 | 0.94 | -3.4 | 55.2 | -1.29 | 0.198 |

| 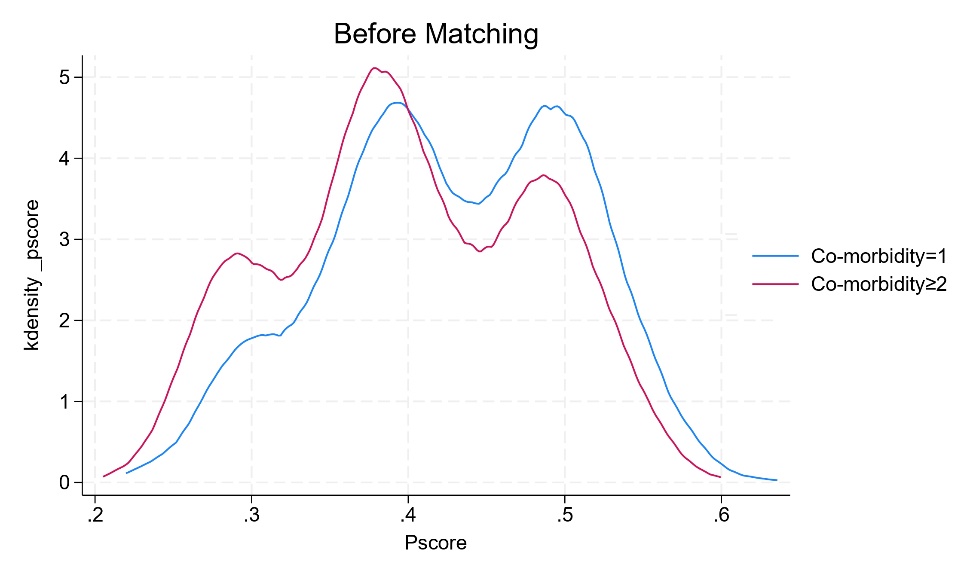 | 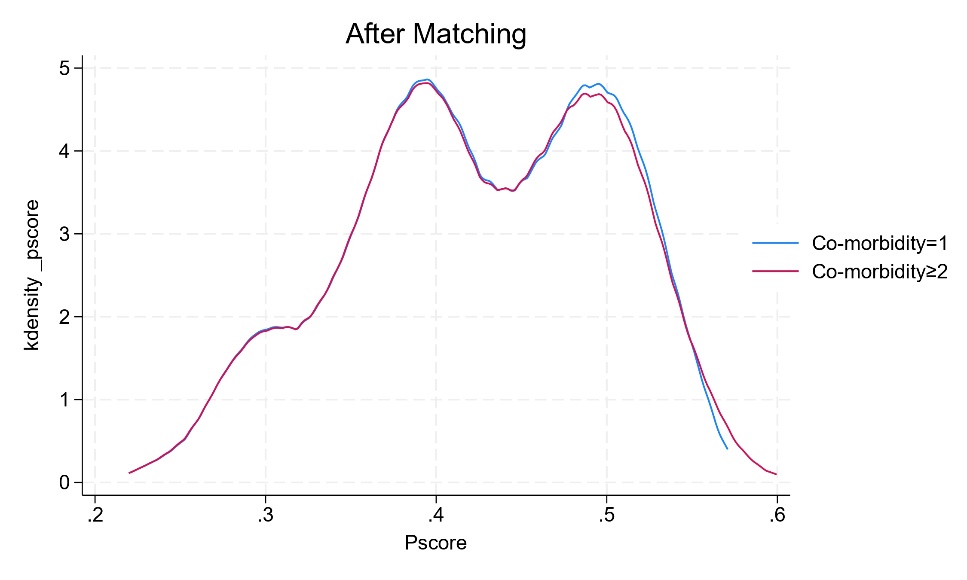 |
| --- | --- |
| **Fig.S3**. Common support domain of PSM for one co-morbidity and two or more co-morbidity groups | |

| **Table.S11.** Differences in lifestyle among co-morbidity status after PSM | | | | |
| --- | --- | --- | --- | --- |
| **Different Lifestyles** | **Total (%)** | **Co-morbidity status** | | ***p*** |
|  |  | Co-morbidity=1 | Co-morbidity≥2 |  |
| **Physical activity** |  |  |  | <0.001 |
| No exercise | 1410 (29.06) | 573 (23.62) | 837 (34.50) |  |
| Exercise | 3442 (70.94) | 1853 (76.38) | 1589 (65.50) |  |
| **Sleep** |  |  |  | <0.001 |
| <7 hour/>8 hour | 1913 (39.43) | 843 (34.75) | 1070 (44.11) |  |
| 7-8 hours | 2939 (60.57) | 1583 (65.25) | 1356 (55.89) |  |
| **Social participation** |  |  |  | <0.001 |
| No | 2355 (48.54) | 1052 (43.36) | 1303 (53.71) |  |
| Yes | 2497 (51.46) | 1374 (56.64) | 1123 (46.29) |  |
| **Lifestyle category** |  |  |  | <0.001 |
| Lifestyle scores<3 | 1874 (38.62) | 786 (32.40) | 1088 (44.85) |  |
| Lifestyle scores≥3 | 2978 (61.38) | 1640 (67.60) | 1338 (55.15) |  |

| **Table.S12.** Subgroup analysis by sex (fully adjusted model) | | | | | | | | |
| --- | --- | --- | --- | --- | --- | --- | --- | --- |
| **Variables** | **Total (%)** | **Physical co-morbidity** | **Psychological co-morbidity** | **Cognitive co-morbidity** | **P-Ps co-morbidity** | **P-C co-morbidity** | **Ps-C co-morbidity** | **P-Ps-C co-morbidity** |
| **Male** | 2187(100.00) |  |  |  |  |  |  |  |
| ***Physical activity*** |  |  |  |  |  |  |  |  |
| No exercise | 745 (34.06) | Reference | Reference | Reference | Reference | Reference | Reference | Reference |
| Exercise | 1442 (65.94) | 0.68(0.55-0.84) | 0.78(0.63-0.96) | 0.48(0.40-0.59) | 0.66(0.53-0.83) | 0.46(0.37-0.57) | 0.50(0.37-0.68) | 0.46(0.33-0.64) |
| ***Diet*** |  |  |  |  |  |  |  |  |
| Unhealthy | 1700 (77.73) | Reference | Reference | Reference | Reference | Reference | Reference | Reference |
| Healthy | 487 (22.27) | 1.15(0.91-1.45) | 0.64(0.50-0.83) | 0.78(0.62-0.99) | 0.63(0.47-0.84) | 0.82(0.64-1.06) | 0.78(0.53-1.14) | 0.74(0.48-1.13) |
| ***Smoking*** |  |  |  |  |  |  |  |  |
| Current | 1197 (54.73) | Reference | Reference | Reference | Reference | Reference | Reference | Reference |
| Never | 990 (45.27) | 1.07(0.89-1.30) | 0.88(0.72-1.08) | 1.15(0.95-1.40) | 0.95(0.76-1.18) | 1.15(0.93-1.41) | 0.98(0.73-1.32) | 1.01(0.73-1.40) |
| ***Sleep*** |  |  |  |  |  |  |  |  |
| <7 hour/>8 hour | 782 (35.76) | Reference | Reference | Reference | Reference | Reference | Reference | Reference |
| 7-8 hours | 1405 (64.24) | 0.91(0.74-1.11) | 0.70(0.57-0.86) | 0.74(0.61-0.90) | 0.67(0.53-0.83) | 0.73(0.59-0.90) | 0.72(0.53-0.97) | 0.69(0.49-0.95) |
| ***Social participation*** |  |  |  |  |  |  |  |  |
| No | 1050 (48.01) | Reference | Reference | Reference | Reference | Reference | Reference | Reference |
| Yes | 1137 (51.99) | 0.67(0.55-0.82) | 0.71(0.58-0.87) | 0.57(0.47-0.69) | 0.68(0.55-0.85) | 0.53(0.43-0.66) | 0.43(0.31-0.59) | 0.46(0.32-0.65) |
| ***Lifestyle category*** |  |  |  |  |  |  |  |  |
| Lifestyle scores<3 | 1173 (53.64) | Reference | Reference | Reference | Reference | Reference | Reference | Reference |
| Lifestyle scores≥3 | 1014 (46.36) | 0.69(0.57-0.83) | 0.63(0.52-0.78) | 0.61(0.50-0.74) | 0.56(0.45-0.71) | 0.56(0.45-0.69) | 0.48(0.35-0.67) | 0.47(0.33-0.67) |
| **Female** | 3870(100.00) |  |  |  |  |  |  |  |
| ***Physical activity*** |  |  |  |  |  |  |  |  |
| No exercise | 986 (25.48) | Reference | Reference | Reference | Reference | Reference | Reference | Reference |
| Exercise | 2884 (74.52) | 0.80(0.67-0.96) | 0.77(0.66-0.89) | 0.57(0.49-0.67) | 0.79(0.67-0.92) | 0.58(0.49-0.68) | 0.59(0.48-0.72) | 0.61(0.49-0.76) |
| ***Diet*** |  |  |  |  |  |  |  |  |
| Unhealthy | 3156 (81.55) | Reference | Reference | Reference | Reference | Reference | Reference | Reference |
| Healthy | 714 (18.45) | 1.02(0.84-1.24) | 0.81(0.68-0.96) | 0.82(0.68-0.98) | 0.87(0.72-1.04) | 0.82(0.67-1.00) | 0.68(0.52-0.89) | 0.68(0.52-0.91) |
| ***Smoking*** |  |  |  |  |  |  |  |  |
| Current | 51 (1.32) | Reference | Reference | Reference | Reference | Reference | Reference | Reference |
| Never | 3819 (98.68) | 1.33(0.74-2.49) | 0.91(0.52-1.62) | 1.11(0.59-2.08) | 0.97(0.53-1.77) | 2.36(1.00-5.60) | 0.79(0.36-1.70) | 1.14(0.45-2.91) |
| ***Sleep*** |  |  |  |  |  |  |  |  |
| <7 hour/>8 hour | 1551 (40.08) | Reference | Reference | Reference | Reference | Reference | Reference | Reference |
| 7-8 hours | 2319 (59.92) | 0.91(0.78-1.06) | 0.64(0.56-0.73) | 0.82(0.71-0.94) | 0.65(0.57-0.75) | 0.75(0.64-0.87) | 0.63(0.52-0.76) | 0.58(0.48-0.71) |
| ***Social participation*** |  |  |  |  |  |  |  |  |
| No | 1830 (47.29) | Reference | Reference | Reference | Reference | Reference | Reference | Reference |
| Yes | 2040 (52.71) | 1.02(0.87-1.18) | 0.76(0.66-0.87) | 0.66(0.57-0.76) | 0.76(0.66-0.87) | 0.65(0.56-0.76) | 0.60(0.49-0.72) | 0.60(0.49-0.74) |
| ***Lifestyle category*** |  |  |  |  |  |  |  |  |
| Lifestyle scores<3 | 1184 (30.59) | Reference | Reference | Reference | Reference | Reference | Reference | Reference |
| Lifestyle scores≥3 | 2686 (69.41) | 0.92(0.78-1.09) | 0.66(0.58-0.77) | 0.61(0.53-0.71) | 0.70(0.60-0.81) | 0.60(0.51-0.70) | 0.52(0.43-0.63) | 0.52(0.42-0.64) |
| Note: P-Ps co-morbidity for physical-psychological co-morbidity; P-C co-morbidity for physical-cognitive co-morbidity; Ps-C co-morbidity for psychological-cognitive co-morbidity; P-Ps-C co-morbidity for physical-psychological-cognitive co-morbidity. Crude OR refer to no adjustments Adjusted OR incorporated sex, age, education, marital status, occupation, disease duration, complication, medication use, and BMI. | | | | | | | | |

**Table.S13.** Sensitivity analysis by excluding over 80 years old

| **Co-morbidity=1** | OR(95%CI) | *P* | aOR(95%CI) | *P* |
| --- | --- | --- | --- | --- |
| 0 | Reference |  | Reference |  |
| 1 | 0.92(0.57-1.48) | 0.734 | 0.78(0.48-1.26) | 0.312 |
| 2 | 0.74(0.47-1.17) | 0.202 | 0.60(0.38-0.95) | 0.031 |
| 3 | 0.72(0.46-1.14) | 0.164 | 0.57(0.36-0.90) | 0.016 |
| 4 | 0.64(0.40-1.01) | 0.053 | 0.47(0.30-0.76) | 0.002 |
| 5 | 0.46(0.24-0.87) | 0.017 | 0.37(0.20-0.72) | 0.003 |
| **Co-morbidity≥2** |  |  |  |  |
| 0 | Reference |  | Reference |  |
| 1 | 0.90(0.49-1.64) | 0.732 | 0.65(0.35-1.22) | 0.18 |
| 2 | 0.68(0.38-1.22) | 0.195 | 0.46(0.25-0.84) | 0.011 |
| 3 | 0.49(0.28-0.88) | 0.017 | 0.31(0.17-0.57) | <0.001 |
| 4 | 0.34(0.19-0.62) | <0.001 | 0.20(0.11-0.37) | <0.001 |
| 5 | 0.09(0.02-0.42) | 0.002 | 0.06(0.01-0.29) | <0.001 |
| **Physical co-morbidity** |  |  |  |  |
| 0 | Reference |  | Reference |  |
| 1 | 0.66(0.35-1.26) | 0.206 | 0.59(0.31-1.14) | 0.115 |
| 2 | 0.58(0.31-1.08) | 0.084 | 0.51(0.27-0.95) | 0.035 |
| 3 | 0.51(0.28-0.96) | 0.036 | 0.44(0.24-0.84) | 0.012 |
| 4 | 0.50(0.27-0.94) | 0.032 | 0.41(0.22-0.78) | 0.007 |
| 5 | 0.70(0.32-1.55) | 0.377 | 0.59(0.26-1.31) | 0.194 |
| **Psychological co-morbidity** |  |  |  |  |
| 0 | Reference |  | Reference |  |
| 1 | 1.26(0.77-2.06) | 0.365 | 0.91(0.55-1.50) | 0.7 |
| 2 | 1.04(0.64-1.68) | 0.88 | 0.67(0.41-1.10) | 0.112 |
| 3 | 0.93(0.58-1.49) | 0.748 | 0.56(0.34-0.91) | 0.02 |
| 4 | 0.72(0.45-1.17) | 0.186 | 0.39(0.24-0.65) | <0.001 |
| 5 | 0.37(0.18-0.76) | 0.007 | 0.23(0.11-0.49) |  |
| **Cognitive co-morbidity** |  |  |  |  |
| 0 | Reference |  | Reference |  |
| 1 | 0.65(0.41-1.05) | 0.076 | 0.56(0.35-0.92) | 0.021 |
| 2 | 0.46(0.29-0.72) | 0.001 | 0.38(0.24-0.61) | <0.001 |
| 3 | 0.36(0.23-0.57) | <0.001 | 0.29(0.18-0.46) | <0.001 |
| 4 | 0.29(0.18-0.47) | <0.001 | 0.23(0.14-0.37) | <0.001 |
| 5 | 0.19(0.10-0.40) | <0.001 | 0.16(0.08-0.33) | <0.001 |
| **P-Ps co-morbidity** |  |  |  |  |
| 0 | Reference |  | Reference |  |
| 1 | 1.09(0.66-1.82) | 0.733 | 0.78(0.46-1.31) | 0.34 |
| 2 | 0.90(0.55-1.48) | 0.679 | 0.57(0.34-0.95) | 0.032 |
| 3 | 0.80(0.49-1.31) | 0.371 | 0.47(0.29-0.79) | 0.004 |
| 4 | 0.63(0.38-1.04) | 0.072 | 0.34(0.20-0.57) | <0.001 |
| 5 | 0.34(0.16-0.73) | 0.006 | 0.21(0.09-0.46) | <0.001 |
| **P-C co-morbidity** |  |  |  |  |
| 0 | Reference |  | Reference |  |
| 1 | 0.63(0.39-1.02) | 0.058 | 0.53(0.33-0.87) | 0.013 |
| 2 | 0.46(0.29-0.73) | 0.001 | 0.38(0.24-0.61) | <0.001 |
| 3 | 0.35(0.22-0.56) | <0.001 | 0.28(0.17-0.45) | <0.001 |
| 4 | 0.27(0.17-0.44) | <0.001 | 0.21(0.13-0.34) | <0.001 |
| 5 | 0.15(0.07-0.34) | <0.001 | 0.12(0.05-0.27) | <0.001 |
| **Ps-C co-morbidity** |  |  |  |  |
| 0 | Reference |  | Reference |  |
| 1 | 0.90(0.49-1.64) | 0.732 | 0.65(0.35-1.22) | 0.18 |
| 2 | 0.68(0.38-1.22) | 0.195 | 0.46(0.25-0.84) | 0.011 |
| 3 | 0.49(0.28-0.88) | 0.017 | 0.31(0.17-0.57) | <0.001 |
| 4 | 0.34(0.19-0.62) | <0.001 | 0.20(0.11-0.37) | <0.001 |
| 5 | 0.09(0.02-0.42) | 0.002 | 0.06(0.01-0.29) | <0.001 |
| **P-Ps-C co-morbidity** |  |  |  |  |
| 0 | Reference |  | Reference |  |
| 1 | 0.91(0.47-1.76) | 0.777 | 0.65(0.33-1.27) | 0.203 |
| 2 | 0.75(0.40-1.42) | 0.377 | 0.49(0.25-0.95) | 0.035 |
| 3 | 0.54(0.29-1.01) | 0.056 | 0.33(0.17-0.64) | 0.001 |
| 4 | 0.35(0.18-0.67) | 0.002 | 0.19(0.10-0.39) | <0.001 |
| 5 | 0.06(0.01-0.48) | 0.008 | 0.04(0.00-0.31) | 0.002 |
| **Note:** P-Ps co-morbidity for physical-psychological co-morbidity; P-C co-morbidity for physical-cognitive co-morbidity; Ps-C co-morbidity for psychological-cognitive co-morbidity; P-Ps-C co-morbidity for physical-psychological-cognitive co-morbidity. Adjusted OR incorporated sex, age, education, marital status, occupation, disease duration, complication, medication use, and BMI. | | | | |
